# Three-Dimensional Modeling of *Camelus dromedarius* T Cell Receptor Gamma (TRG)_Delta (TRD)/CD1D Complex Reveals Different Binding Interactions Depending on the TRD CDR3 Length

**DOI:** 10.3390/antib14020046

**Published:** 2025-05-29

**Authors:** Salvatrice Ciccarese, Marie-Paule Lefranc, Giulia C. M. Perrone, Pietro D’Addabbo, Ciro Leonardo Pierri

**Affiliations:** 1 Department of Biosciences, Biotechnologies and Environment, University of Bari Aldo Moro, Via E.Orabona, 4-70125 Bari, Italy; pietro.daddabbo@uniba.it; 2 IMGT^®^, the International ImMunoGeneTics Information System^®^ (IMGT), Laboratoire d’ImmunoGénétique Moléculaire (LIGM), Institut de Génétique Humaine (IGH), Centre National de la Recherche Scientifique (CNRS), Université de Montpellier (UM), 34090 Montpellier, France; marie-paule.lefranc@umontpellier.fr; 3 Laboratory of Biochemistry, Structural, and Molecular Biology, Department of Pharmacy—Pharmaceutical Sciences, University of Bari, Via E. Orabona, 4-70125 Bari, Italy; g.perrone42@phd.uniba.it

**Keywords:** T cell receptor, *Camelus dromedarius*, TRG and TRD loci, multiple sequence alignments (MSA), 3D modeling, protein–protein interaction, IMGT unique numbering

## Abstract

Background: In the adaptive immune response of the dromedary (*Camelus dromedarius*, Camdro), the T cell receptor (TR) repertoire of the gamma–delta (γδ) T cells is unusually diversified both by somatic hypermutation in rearranged TR gamma (TRG) and delta (TRD) genes and by the diversity in sequence and length of the third complementarity-determining region (CDR3) of the TRD chain. Methods: The purpose was to investigate, in the absence of 3D structures, the role of Camdro γδ T cells, focusing on the binding interactions at the interface between the V-gamma and V-delta domains, and in complex with the CD1D, a major histocompatibily class I (MH1)-like glycoprotein presenting lipid antigen in association with B2M. A combination of hypermutated TRG dromedary cDNA clones was paired with TRD clones bearing very long, long, or short CDR3s, all isolated from the spleen of a single animal. Results: The 3D models of the Camdro TRG_TRD/CD1D_B2M complexes were inferred using the *Homo sapiens* 3D structure and the ImMunoGeneTics (IMGT) numbering for V, C, and G domains, and investigated for binding interactions at the interface of the paired V-gamma_V-delta and at the interface with CD1D. Our results suggest that transcripts with long CDR3s may derive from a population of CD1D-restricted γδ T cells. Both the CD1D G-alpha1-like and G-alpha-2 like domain helices were contacted by both the V-gamma and V-delta CDR-IMGT loops. Conclusions: Our findings further emphasize the similarity between the γδ T cells population we analyzed in *Camelus dromedarius* and the CD1D-restricted γδ NKT cells in *Homo sapiens*.

## 1. Introduction

The antigen receptors of the adaptive immune responses of jawed vertebrates (*gnathostomata*), from fish to *Homo sapiens*, comprise the immunoglobulins (IGs) or antibodies expressed on the membrane of B cells and secreted by the plasma cells and the T cell receptors (TRs) alpha–beta (αβ) and gamma–delta (γδ) expressed on the membrane of T cells [1]. The IGs recognize antigen in their native (unprocessed) form [2,3], whereas the TRs alpha–beta recognize processed antigens, which are presented as peptides by the highly polymorphic major histocompatibility (MH, in humans HLA for leukocyte antigens) proteins [4]. In contrast, the ligands bound by the TR gamma–-delta are more diverse and are presented by related proteins of the immune (RPI) –MH1-like proteins such as CD1C, CD1D, and MR1 (Figure S1) [5,6]. The chains of the IG and TR αβ and γδ antigen receptors are characterized by a variable domain at their N-terminal end (1). The synthesis of these variable domains results from the rearrangements of variable (V), diversity (D), and joining (J) genes, which create highly diversified V– (D) –J repertoires of 10^12^ antigen receptors in each individual [2,4]. The VH domains of the IG heavy (IGH) chains, the V-beta domain of the TR beta (TRB) and the V-delta domain of the TR delta (TRD) chains result from V–D–J rearrangements (with a usual number of rearranged D genes per junction from one to three, depending on the locus), whereas the VL domains of the IG light kappa (IGK) and lambda (IGL) chains (V-kappa and V-lambda in the higher vertebrates) and iota (IGI) chains (V-iota in the lower vertebrates), the V-alpha of the TR alpha (TRA), and the V-gamma of the TR gamma (TRG) result from V-J rearrangements [1]. To deal with the huge repertoires, the immense data diversity and the specific characteristics of the jawed vertebrate’s adaptive immune responses, a new science, immunoinformatics, has emerged at the interface between immunogenetics and bioinformatics [1], with the founding of IMGT in 1989 by Marie-Paule Lefranc. IMGT databases, tools, and Web resources have been built on the IMGT-ONTOLOGY concepts and on the IMGT Scientific chart rules with, among them, the IMGT Nomenclature [7,8,9,10,11] and the IMGT unique numbering [12,13] having become the two pillars of immunoinformatics. A major characteristic of the IMGT numbering is to bridge genes, sequences, structures, and functions of the V-domain [12], of the C-domain [13], and of the G-domain [14]. The V-domain (IMGT keyword) includes the V-DOMAIN (IMGT label in capital letters) of the jawed vertebrates IG and TR antigen receptors for which they were created but also the V-like domains of the immunoglobulin superfamily (IgSF) members other than IG and TR, whatever the species [12]. Similarly, the C-domain includes the C-DOMAIN of the jawed vertebrates IG and TR antigen receptors, and the C-like domains of the IgSF members other than IG or TR, whatever the species [13]. The G-domain includes the G-DOMAIN of the jawed vertebrates MH and the G-like domains of the MH superfamily (MhSF) members other than MH, whatever the species [14]. The IMGT unique numbering for V-domain, C-domain, and G-domain has been used for many years for the IG/antigens and TR alpha-beta TRA_TRB/pMH complexes [15,16,17,18,19,20,21,22,23,24,25,26,27,28,29,30] and for the TR gamma–delta/non peptidic antigen [31,32,33,34] in the IMGT/3Dstructure-DB database [35,36,37], the IMGT/DomainGapAlign tool being used for the amino acid sequences analysis [38,39] and the IMGT Colliers de Perles for the domain graphical representations [40,41] or the visualization of amino acids polymorphisms or engineered variants [42]. The 3D structures of the TRG_TRD/nonpeptidic antigens complexes are still rare, and 3D computational modeling creates an opportunity for predicting binding interactions for species such as *C. dromedarius*. In the context of the cellular-mediated response of the adaptive immunity, camels are like other artiodactyls, such as sheep, cows, and pigs, with higher frequencies of blood γδ T cells (up to 35% of T cells) in younger animals. Therefore, camels belong to the “γδ-high species”, in contrast to “γδ-low species”, like humans and mice, where γδ T cells represent only a minor subpopulation (<5%) of circulating lymphocytes. As in the other species, the Camdro γδ T cells are characterized by the expression at their surface of the T cell receptor (TR), a membrane heterodimer made of a TR gamma (TRG) chain and a TR delta (TRD) chain. The V-gamma domain is encoded by rearranged TRGV–TRGJ genes [43] and the V-delta domain by rearranged TRDV–(TRDD)–TRDJ genes [44]. It is still unclear how γδ T cells use their V–(D)–J rearranged T cell receptor (TR) to recognize nonpeptide antigen in an antibody-like fashion or presented by RPI–MH1-like proteins. Interestingly, compared to other species, camels possess unusual features in the immune system [45,46,47,48]. The rearranged V-gamma and V-delta domains of the *C. dromedarius* TRG_TRD receptor display somatic hypermutations (SHM) that enhance the γδ T cell repertoire diversity [46]. Moreover, an unusual expansion and diversity of the complementarity-determining region 3 (CDR3-IMGT) of the V-delta domain results from multiple TRDD genes involved in the V–D–J rearrangement which enables these cells to participate in a broader range of antigen recognition, hinting at complex interactions with nonpeptide antigens [44,45,46,49]. We have recently identified CD1D-restricted γδ T cell receptor in dromedaries from three-dimensional structure modeling inferred from *C. dromedarius* cDNA clones [50,51]. Here, we analyze the protein–protein interaction in a TRG_TRD/CD1D complex selected for the camel-specific characteristics of its antigen receptors (long CDR3-IMGT, somatic mutations), using the IMGT numbering of the V, C, and G domains as a paradigm for bridging amino acid sequences, 3D models, structures, and functions between species.

## 2. Materials and Methods

### 2.1. Crystal Structure Sampling Through Fold Recognition, Multiple Sequence Alignments (MSA), and 3D Modeling of C. dromedarius TRG and TRD in Complex with C. dromedarius CD1D Antigen and B2M

Fold recognition methods implemented in pGenThreader and i-TASSER were used to identify homologous protein-crystallized structures for TRG and TRD chains. The amino acid sequences of *Camelus dromedarius* TRG (AFD98894.1 and AFD98918.1; accession JF755952.1 and JF792640.1) and TRD (CAX52224.1, CAX52229.1, and CAX52219.81; accession FN252371.1, FN252376.1, and FN252345) proteins (in the FASTA format) were used as query sequences in pGenThreader (http://bioinf.cs.ucl.ac.uk/psipred/, accessed on 4 March 2025) and i-TASSER (https://zhanglab.ccmb.med.umich.edu/I-TASSER/, accessed on 4 March 2025) to search the PDB for the most similar deposited crystallized structures [52,53,54,55,56]. SPDBV was then used to build 3D all-atom models of the *C. dromedarius* TRG and TRD chains, using the *H. sapiens* TRG and TRD structures from the crystallized TRG_TRD receptor in complex with CD1D antigen and B2M chains, available under the PDB code “4lhu.pdb” [51].

The *H. sapiens* CD1D antigen and B2M chain sequences were used as query sequences in BLASTp to identify their counterparts in *C. dromedarius*. The identified sequences were modeled using the *H. sapiens* CD1D antigen and B2M chain structures from PDB code “4lhu.pdb” as templates, following our validated protocols [55,56,57].

All generated 3D all-atom models were then energy-minimized using the Yasara Minimization server [56,57,58].The proposed 3D comparative modeling of *C. dromedarius* TRG and TRD chains in complex with *C. dromedarius* CD1D antigen and B2M chains was generated by aligning the individual *C. dromedarius* domains (TRG, TRD, CD1D antigen, and B2M) with the corresponding atomic coordinates of each chain from the crystallized *H. sapiens* TRG_TRD receptor in complex with the *H. sapiens* CD1D and B2M chains, available under PDB code 4lhu.pdb [51].

The obtained 3D models of *C. dromedarius* TRG and TRD chains were superimposed onto the crystallized structures of *H. sapiens* TRG and TRD from “4lhu.pdb” [51]. Similarly, the 3D models of *C. dromedarius* CD1D antigen and B2M chains were superimposed onto their respective *H. sapiens* counterparts in the 4lhu.pdb crystallized structure.

Superimposition operations were carried out using the “super” command in PyMOL, beginning with the structural alignment of the analyzed backbones. The “super” command enables the alignment of selected proteins for comparative structural analysis by providing a sequence-independent, structure-based pairwise alignment. Notably, it is more robust than the “align” command, as it can successfully superimpose proteins with lower sequence similarity, as observed in previous applications [54,56,57,59,60,61,62,63,64]. Following this, missing or buried residues at the protein–protein interface were modeled and relaxed to resolve clashes and potential breaks in the backbone structure [50,54,55,57,65]. All generated 3D all-atom models were then energy-minimized using the Yasara Minimization server, as described for previous applications [50,54,55,57,58,65,66,67,68,69,70]. The final models were visually inspected in VMD, PyMOL, and SPDBV to identify any remaining unsolved clashes [50,54,55,57,58,65,66,67,68,69,70]. The structural quality of the generated 3D models was evaluated using MolProbity, QMEANDisCo (via the SWISS-MODEL Workspace: https://swissmodel.expasy.org/assess, accessed on 4 March 2025) and Verify3D (via the SAVES platform: https://saves.mbi.ucla.edu/, accessed on 4 March 2025). Protein–protein binding regions were identified by selecting residues within 4 Å at the protein–protein interface in the superimposed structures.

### 2.2. FoldX Energy Calculations

The FoldX AnalyseComplex assay was used to calculate the interaction energies between the three generated *C. dromedarius* TRG and TRD models, as well as between each of the six combined TRG_TRD receptors in complex with the *C. dromedarius* CD1D antigen and B2M chains. Additionally, it was employed to determine the interaction energy for the *H. sapiens* counterparts from the crystallized 4lhu.pdb, which served as a reference system for validation and comparative purposes.

FoldX operates by unfolding the selected targets, determining the stability of the remaining molecules, and then subtracting the sum of the individual energies from the total energy. More negative energy values indicate stronger binding, while positive values suggest no binding, as described in previous applications [57,65,71,72,73,74,75,76,77,78]. FoldX also provides a decomposition of the total interaction energy into individual physical components, including van der Waals forces, electrostatic interactions, hydrogen bonding, solvation effects, and entropic penalties. These are reported on a per-residue basis but not at the level of individual atom–atom pairs. However, these estimations offer a compromise between interpretability and computational efficiency, enabling the identification of key residues driving binding affinity. All FoldX calculations were performed using FoldX 5.0 (https://foldxsuite.crg.eu/products#foldx, accessed on 4 March 2025) and default parameters.

## 3. Results

### 3.1. Identification and Selection of Dromedary TRD cDNA Clones Having a Long CDR3

**Figure 1:**
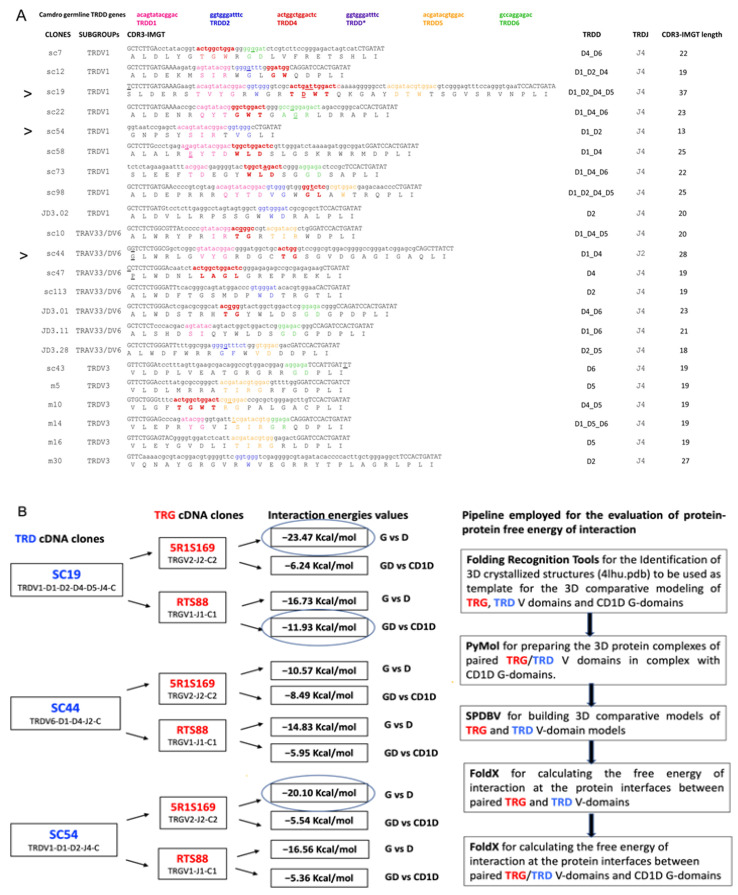
(**A**) CDR3-IMGT nucleotide (nt) and predicted amino acid (AA) sequences retrieved from the TRD cDNA clones isolated from spleen [44]. CDR3-IMGT sequences are shown from codon 105 (the codon after the 2nd-CYS 104 of the V-REGION) to codon 117 (the codon before J-PHE 118 of the J-REGION) according to the unique numbering for V domain [12]. The CDR3 nucleotide/amino acid sequence, and the classification of the TRDV and TRDJ genes of each clone are also listed. The TRDV genes were assigned to the corresponding germline subgroup only [48]. Nucleotides of the 3’V-REGION and of the 5′J-REGION are indicated in uppercase letters. The sequences considered to present recognizable TRDD genes are indicated in colored lowercase letters (pink for TRDD1, blue for TRDD2, red for TRDD3, orange for TRDD5, green for TRDD6, and dark purple for the TRDD, missing in the current assembly), and the nucleotide substitutions are underlined. The amino acids belonging to a TRDD gene are in bold. Nucleotides that cannot be attributed to any V, D, or J region (P/N-nucleotides) are indicated in lower cases on the left and on the right sides of the TRDD regions. The name of each clone is reported with CDR3-IMGT length (AA), and with the contribution of TRD 3′V-REGION, TRDD, TRDJ 5′J-REGION, and P/N nucleotide sequences, using colors, small and capital letters as described above. (**B**) Scheme of the pipeline employed for the evaluation of protein–protein interaction energies. On the left, each TRD (SC19, SC44 and SC54) cDNA clone was combined with each TRG (5R1S169 and RTS88) cDNA clones. The V-(D)-J-C gene composition of the Camdro paired TRG_TRD V-domains corresponding to each combination is provided. In correspondence with each combination of the TRG and TRD examined clones (black arrows), the estimated negative energy values (kcal/mol) of the interactions at the protein interface in the complex between the paired TRG and TRD V-domains, and at the protein interfaces between the paired TRG/TRD V-domains and the CD1D G-domains are shown in succession. Additionally, the V-(D)-J-C gene composition of the paired TRG/TRD V-domains corresponding to each combination is provided. G vs. D indicates the value in Kcal/Mol referring to the dimer TRG_TRD; GD vs. CD1D indicates the value referred to the complex (TRG_TRD)/CD1D. The values inside the circles are considered the most significant, as they represent those closest to the H. sapiens negative energy value (see Table S1).

With the aim of calculating the most likely interactions at the protein interface between the TRG_TRD heterodimer in complex with *C. dromedarius* CD1D, TRD clones SC19, SC54, SC44 isolated from the spleen of a single adult individual (Figure 1A,B) were chosen for pairing with the RTS88 and/or 5R1S1S9 hypermutated TRG clones [46], the latter isolated from the spleen of the same animal.

In previous research, nucleotide changes relative to the germline sequences were identified by analyzing 39 cDNA clones (9 in spleen, 8 in tonsils and 22 in blood) consisting of a member of the TRDV1 subgroup, 11 clones (7 in spleen and 4 in tonsils) containing the TRAV33/DV6 genes, and 11 clones (6 in spleen, 3 in tonsils, and 2 in blood) retaining the TRDV3 gene [48].

In addition, the contribution of each annotated germline gene in the formation of the TRD chain repertoire was evaluated with reference to the sequence and length of the CDR3-IMGT. A total of 61 cDNA clones isolated from peripheral lymphoid tissues of an adult healthy dromedary (22 from spleen, 15 from tonsils, and 24 from blood) and containing unique rearranged productive V–(D)–J–C transcripts, were selected from the cDNA library [44] and analyzed for the identification of enlarged CDR3-IMGT.

For a close inspection of the CDR3-IMGT, the nucleotide sequences and translation from codon 105 (codon following the 2nd-CYS codon 104 of the V-REGION) to codon 117 (codon preceding the J–PHE 118 or J–TRP 118) that belongs at the J-MOTIF F/W–G–X–G motif characteristic of the J–REGION (i.e., coding region of a J gene) are reported in Figure 1A and Figures S2 and S3. The obtained CDR3-IMGT length (AA) mean value ranges are, respectively, 13–37 in the 22 from spleen clones (Figure 1A), 12–32 in the 24 from blood clones (Figure S2), and 15–32 in the 15 from tonsils clones (Figure S3).

### 3.2. Combination of C. dromedarius TRG and TRD cDNA Clones and 3D Modeling of the Paired V-Gamma and V-Delta Domains in Complex with the CD1D G-Domains and B2M

*C. dromedarius* TRG (TR gamma) and TRD (TR delta) chains were modeled by using as a protein template the *H. sapiens* TRG (4lhu.pdb) chain, sharing with the *C. dromedarius* a TRG chain coded by RTS88 or 5R1S169 clones, 47 or 41% of identical amino acids, respectively, and the human TR delta (4lhu.pdb) chain, sharing with *C. dromedarius* a TRD chain coded by SC19, SC54, or SC44 clones, 54, 62, or 44% of identical amino acids, respectively. Remarkably, *C. dromedarius* TRD share more than 48% of identical residues (SC44. vs. SC19 and SC44. vs. SC54) till to 68% for SC54. vs. C19). The percentages of identical residues shared between the sequences to be modeled and the human crystallized TRGD were coherent with percentages of identical residues expected for performing comparative modeling [79,80]. The V-domain pairwise sequence structure alignment between the investigated *C. dromedarius* TRG and TRD and the *Homo sapiens* crystallized structure with PDB code 4LHU generated by ClustalW [81,82] (Figure S4A,B) was reproduced into the SPDBV alignment panel to guide and build the 3D comparative model of both TR V-domains, following validated protocols [52,54,55,56,83].

The quality of the generated 3D models was evaluated using MolProbity, QMEANDisCo, and Verify3D [84,85,86,87,88]. All models showed a MolProbity score below 2.3, a Clash Score under 6, more than 93% of residues in Ramachandran favored regions, and less than 1.3% Ramachandran outliers. QMEANDisCo scores were above 0.7. Verify3D analysis indicated that between 83% and 90% of residues had an averaged 3D-1D score ≥ 1. These results fall within generally accepted thresholds for reliable, high-quality protein models [84,85,86,87,88] (Figure S5). The scheme of the pipeline employed for the evaluation of protein–protein interaction energies is shown in Figure 1B.

To investigate the interactions between the TRG and TRD V-domains and the G domains of CD1D in *Camelus dromedarius*, we searched for the corresponding CD1D sequence in this species and identified XP_031291871.1. This sequence shares over 71% amino acid identity with the human CD1D (4LHU) and exhibits 100% sequence coverage (Figure S4C). Similarly, to identify the closest B2M homolog in *C. dromedarius*, we found the sequence XP_031309022.1, which shows more than 78% identity and full coverage when compared to the human B2M sequence (Figure S4D).

Starting from the alignments of the sequences taken from 4lhu.pdb, a 3D comparative model of the *C. dromedarius* CD1D and B2M was built. The alpha carbon (Ca) root mean square deviation (RMSD) between the coordinates of the built 3D comparative models and the crystal structure with PDB code 4LHU ranged between 0.58 and 0.61 Å.

In terms of interaction energies calculated by FoldX, the strongest interactions between the investigated *C. dromedarius* TRG and TRD 3D models of the V-domains are observed in the 5R1S169_SC19 (−23.47 kcal/mol) which has the longer TRD CDR3-IMGT (37 AA for SC19) compared to all the TRG_TRD combinations tested and which corresponds to the pairing TRGV2-J2-C2_TRDV1-D1-D2-D4-D5-J4-C and in the 5R1S169_SC54 (−20.10 kcal/mol), which corresponds to the pairing TRGV2-J2-C2_TRDV1-D1-D2-J4-C (Figure 1B, Table S1).

Furthermore, the strongest interactions between the investigated *C. dromedarius* TRG/TRD 3D models of the V-domains and CD1D are observed in the RTS88_SC19/CD1D (−11.19 kcal/mol) which corresponds to the pairing TRGV1-J1-C1_TRDV1-D1-D2-D4-D5-J4-C. Meanwhile, less strong interactions are observed both in the RTS88_SC44/CD1D (−5.95 kcal/mol) which corresponds to the pairing TRGV1-J1-C1_TRDV6-D1-D4-J2-C and in the RTS88_SC54/CD1D (−5.36 kcal/mol) which corresponds to the pairing TRGV1-J1-C1_TRDV1-D1-D2-J4-C (Figure 1B, Table S1).

### 3.3. Involvement of the Long TRD CDR3 in the CD1D-Restriction of γδ T Cell in Dromedaries

**Figure 2:**
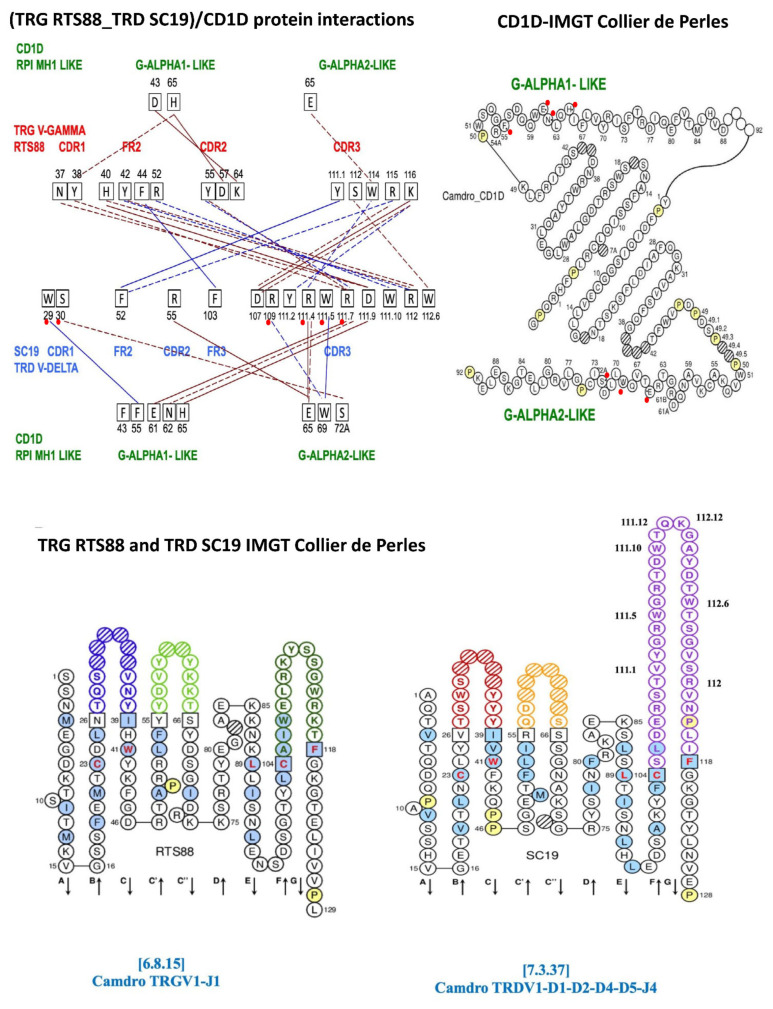
Protein–protein interactions at the protein interface between the *Camelus dromedarius* (Camdro)-paired TRG V-GAMMA (clone RTS88) and TRD V-DELTA (clone SC19) V-domains and at the protein interface in the complex between the paired TRG_TRD V-domains and the Camdro CD1D G-ALPHA1-LIKE and G-ALPHA2-LIKE G-domains. Four types of protein–protein interactions are shown: Protein–protein Ionic interactions within 6 Å (solid brown line), protein–protein side chain–side chain hydrogen bonds (dashed brown line), aromatic–aromatic interactions within 4.5 and 7 Å (solid blue line), cation–π interactions (dashed blue line). **Top left**, the amino acids involved in the interactions are shown in black squares with their positions according to the IMGT unique numbering for V-domain [13]. CDR and FR to which belong the AA involved in the interactions are written in red for RTS88 TRG V-GAMMA and in blue for SC19 TRD V-DELTA. In green the CD1D G-ALPHA1-LIKE and the G-ALPHA2-LIKE domains, with their respective amino acids and their positions involved in the interactions with the paired RTS88/SC19 V-domains. **Top right**, IMGT Collier de Perles of the G-ALPHA1-LIKE and the G-ALPHA2-LIKE domains of the Camdro CD1D [14], obtained using the IMGT/Collier-de-Perles tool [36]. Bottom left to right, IMGT Collier de Perles of the Camelus dromedarius TRGV1-J1 V-gamma domain [6.8.15] (clone RTS88) and the TRDV1-D1-D2-D4-D5-J4 V-delta domain [7.3.37] (clone SC19) [12,44,45]. For a list of all the detected interactions, see Supplementary Table S2. This list includes the four types of interaction reported Figure 2 top left and in Table 1, and in addition three types of interactions, hydrophobic interactions within 5 Å, main chain–main chain hydrogen bonds, and main chain–side chain hydrogen bonds. The software specifically used to generate these structural visualizations is part of the IMGT/Collier de Perles toolset, available through the IMGT web platform (https://www.imgt.org/, accessed on 4 March 2025).

Six types of protein–protein interactions, ionic interactions within 6 Å, side chain hydrogen bonds, aromatic–aromatic interactions within 4.5 and 7 Å, aromatic–sulfur interactions, cation–π interactions, hydrophobic interactions within 5 Å, were identified and analyzed (Table S2). These energy contributions are reported on a per-residue basis. Four types of interactions are shown in Figure 2 (top left), which identifies the interacting amino acids at both at the V-gamma_V-delta interface and at the (TRG_TRD)/CD1D interface.

The protein–protein interaction at the interface of the paired *C. dromedarius* (Camdro) V-gamma (V-GAMMA RTS88) and V-delta (V-DELTA SC19) domains are reported in Table 1A. Eighteen interactions involve twelve amino acids of the V-gamma: two of the CDR1 (N37 and Y38), five of the FR2 (H40, Y42, F44, R52, and Y55) and five of the CDR3, and ten amino acids of the V-delta (one of the FR2 F52, one of the FR3 F103, and eight of the CDR3). Four types of interactions are shown, namely: two ionic interactions within 6 Å (FR2 H40/CDR3 D111.9 and CDR3 K116/CDR3 D107), eight side chain–side chain hydrogen bonds interactions (e.g., CDR1 N37/CDR3 D111.9), two aromatic–aromatic interactions within 4.4 and 7 Å (FR2 F44/FR3 F103, CDR3 Y111.1/FR2 F52), and six cation–π interactions (e.g., FR2 Y42/CDR3 R112).

Protein interactions between the V-gamma_V-delta domains of *C. dromedarius* RTS88_SC19 in complex with *C. dromedarius* CD1D are shown in Table 1B. Fourteen interactions involve four amino acids of the V-gamma and eight amino acids of the V-delta in contact with the eight amino acids of CD1D (five amino acids of the G-alpha1-like domain and three amino acids of the G-alpha2-like domain). Except for D43 of the CD1D G-alpha1-like groove floor (Figure 2 top right) contacted by the V-gamma CDR2 K64, the other seven amino acids of the CD1D belong to the helix of the G-domain.

The four types of interactions reported in Table 1B comprise six ionic interactions within 6 Å, two for the V-gamma/CD1D G-alpha1-like (CDR2 D57/H65, CDR2 K64/D43) and four for the V-delta/CD1D comprising two with G-alpha1-like (CDR3 R111.7/E61, CDR3 D111.9/H65) and two with G-alpha2-like (FR2 R55/E65, CDR3 R111.4/E65. Five side chain–side chain hydrogen bonds interactions, two involving V-gamma, one with G-alpha1-like (CDR1 Y38/H65) and one with G-alpha2-like (CDR3 W114/E65), and three involving V-delta, one with G-alpha1-like (CDR3 R111.7/N62) and two with G-alpha2-like (CDR1 S30/S72A, CDR3 R111.4/E65), two aromatic–aromatic interactions within 4.4 and 7Å between V-delta and CD1D, one with G-alpha1-like (CDR1 W29/F55) and one with C-alpha2-like (CDR3 W111.5/W69), and one cation–π interaction (CDR3 R109/W69) are reported in Table 1B.

The high number of interactions between the V-delta CDR3, with both the V-gamma and with the CD1D G-domains correlates with the lower negative energy values in terms of Kcal/mol that are found in the pairings of V-gamma/V-delta domains that include clone SC19 and in complex with CD1D. The values within the circles are regarded as the most relevant, as they are the ones most closely aligned with the human negative energy value (Figure 1B, Table S1).

**Figure 3:**
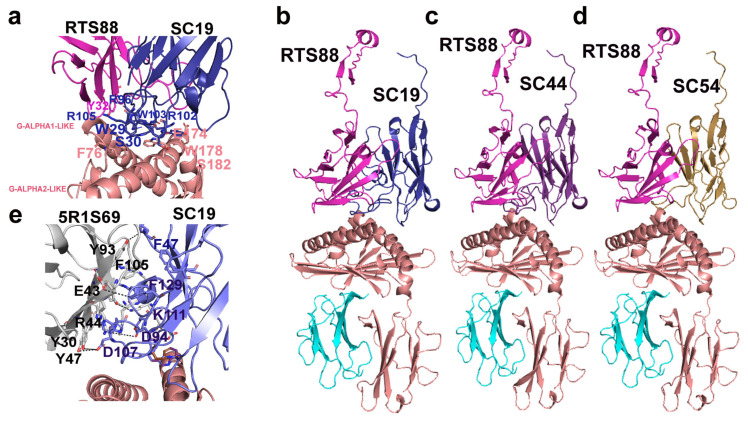
The 3D comparative models of the *C. dromedarius* γδTR in complex with the 3D model of *C. dromedarius* CD1D are reported in cartoon representation. Panel (**a**). A zoomed view of the surface of interaction in the 3D comparative model of the γδTR (coded by RTS88, in magenta, and SC19, in dark blue, respectively) in complex with CD1D (in salmon) highlights examples of interacting amino acids involved in protein–protein interactions (see Table 1). The IMGT numbering is followed by the corresponding PDB numbering in brackets. Corrections: G-ALPHA-1-LIKE: F55 (instead of 76). G-ALPHA-2-LIKE: E65 (instead of 174), S72A (instead of 182), W69 (instead of 178). TRD: W29 and S30: OK, R111.7 (instead of 105), R109 (instead of 96), W111.5 (instead of 103), R111.4 (instead of 102). TRG: Y38 (instead of 32). Panels (**b**–**d**), the 3D comparative models of the indicated γδTR in complex with the 3D model of *C. dromedarius* CD1D are reported for comparative purposes. RTS88 (TRG) is in magenta, SC19 in deep blue, whereas CD1D is in salmon. SC44 and SC54 are reported in pink and light orange, respectively. In the 3D models of panels (**b**–**d**), B2M is in cyan. Panel (**e**). A zoomed view of the surface of interaction in the 3D comparative model of the γδTR (coded by 5R1S69, in gray, and SC19, in blue, respectively) in complex with CD1D (in salmon) is reported in panel (**e**). The negative energy value corresponding to −23.47 Kcal/mol in the 5R1S169_SC19 complex is shown in Figure 1B and in Table S1.

Figure 3 (panel a) highlights a zoomed view of the surface of interaction in the 3D *C. dromedarius*/*H. sapiens* comparative model of the γδ TR (*C. dromedarius* RTS88/SC19) in complex with CD1D for amino acids involved in binding interactions. Remarkably, the three arginine residues R96, R102, R105 (according to PDB numbering) observed in CDR3 of SC19 are deeply involved in a complex network of H-bonds or ionic/cation–π interactions with aromatic, hydrophilic, or basic residues of the γ TR domain. At the same time, the mentioned arginine residues are also involved in ionic/cation–π interactions with acidic/aromatic residues on both G-ALPHA1-LIKE and G-ALPH2-LIKE CD1D domains. Figure 3 (panel e) highlights a zoomed view of the surface of interaction in the 3D *C. dromedarius*/*H. sapiens* comparative model of the γδ TR (*C. dromedarius* 5R1S69/SC19) in complex with CD1D for amino acids involved in binding interactions.

## 4. Discussion

A previous study quantified the interaction strength within the *H. sapiens* γδ T cell repertoire capable of binding to human CD1D [50,51]. This subset of γδ T cells expresses TRGV5–TRGJ1 and TRDV1–(D)–TRDJ1 chains. Furthermore, consistent with the proposed classification of natural killer T cells as γδ NKT cells [89,90], we suggested a potential mechanism by which the γδ TCR of a human NKT cell could interact with the CD1D domains presented by an antigen-presenting cell (APC) [50,51,90,91,92,93].

In this report, to complete what has been described in man, we include a graphical representation of the γδ NKT (Figure S4) and the correspondence between the IMGT DOMAIN numbering, the IMGT file numbering, the PDB numbering for the residues (amino acid three-letter and one-letter abbreviations) of the domains 1. V-GAMMA, 2. V-DELTA, 3. G-ALPHA1-LIKE, and G-ALPHA2-LIKE of the PDB entry 4lh4 (IMGT/3Dstructure-DB card for 4lhU, https://www.imgt.org/3Dstructure-DB/cgi/details.cgi?pdbcode=4LHU, accessed on 4 March 2025, IMGT numbering comparison) (Table S3).

Our discussion focuses on a comparative analysis of the different negative energy values for diverse CDR3 lengths both in the interactions between the variable domains of the TRG/TRD dimer and in the interactions between the dimer itself and the CD1D G-alpha1-like and G-alpha-2 like domain helices. For calculating the free energy of interaction at the TRG–TRD/CD1D protein–protein interface, we modeled TCR chains and built the related protein complexes with CD1D as described above, in line with the previously reported protocols [50,51]. While the employment of a single starting model might indicate an attenuated difference between the different modeled *C.dromedarius* TCR chains, it should be stressed that the mentioned chains share more than 40% of identical residues and till 75% of similar residues, with the crystallized human TCR chains, used as a template for modeling. Considering the shared identical/similar residues, it is expected that the three protein complex models will show a highly similar overall structure. Notably, the obtained complexes were also energetically minimized, before calculating the free energy of interactions at the protein–protein interface, for ensuring the resolution of possible clashes due to the construction of the 3D protein complex models. The structural models were validated using MolProbity, QMEANDisCo, and Verify3D, yielding overall scores, Ramachandran-favored regions, and clash scores compatible with good models [84,85,86,87,88]. By analyzing the results coming from the calculation of the free energy of interaction, it was observed that when the interactions at the TRG/TRD interface become weaker (interaction energy from −23.47 to −16.73 kcal/mol), the longer SC19 (TRD-CDR3) appears to favor stronger interactions at the TRG-TRD/CD1D protein–protein interface (from −6.24 to −11.93). However, it is not simply a matter of the length of TRD-CDR3. Indeed, clone SC44, whose CDR3 is longer than that of clone SC54, causes a stronger interaction energy with CD1D only when the TRG/TRD interactions are relatively weaker (i.e., in the pairing with 5R1S169, −10.57 kcal/mol). Conversely, the shortest SC54 causes the formation of the second strongest interaction at the TRG/TRD interface (−20.10 Kcal/mol), whereas interactions at the TRG-TRD/CD1D protein–protein interface in the presence of the shortest SC54 are slightly weaker (between −5.36 and −5.54 kcal/mol) than those observed in the presence of SC44 (i.e., −5.95 kcal/mol). Thus, in accordance with [94,95,96,97,98], which describe the mechanisms by which protein folding can modify their affinity and specificity for other proteins and how variability can influence interactions with targets (MHC or CD1D) but also the binding specificity towards some antigens, it can be speculated that the length of the TRD-CDR3 can determine a reinforcement of the interactions with CD1D in the presence of weaker interactions at the TRG/TRD protein–protein interface, which can depend on the folding of specific TRG/TRD pairs.

Previous studies have highlighted how protein folding can determine the ability of a protein to interact with other molecules and macromolecules, emphasizing the role of residue interactions at the protein–protein interfaces [95,99,100,101,102]. Additionally, scientific research has explored how changes in folding can alter protein interactions, affecting their stability and efficacy [103,104,105,106].

Through a comparative analysis of binding energies at protein interfaces, our findings are coherent with the existence of a specialized subset of CD1D-restricted γδ T cells in camels, characterized by a highly diverse TRD CDR3. These findings contribute to the growing body of evidence suggesting that camelid γδ T cells possess a distinctive ability to recognize CD1D-presented antigens, likely through a series of interactions mediated by an elongated TRD CDR3. The strong interaction energies and stability observed in our modeled *C. dromedarius* (TRG_TRD)/CD1D complexes suggest a binding mechanism in which the TRD CDR3 loop plays a pivotal role. This unique feature could underpin a broader lipid antigen recognition capacity [97,107,108,109,110], enhancing the immunological versatility of camels. Notably, the structural adaptations we observed in the TRD CDR3 mirror those reported in certain *H. sapiens* γδ TR, particularly within subsets exhibiting autoreactivity to CD1D [89].

Our findings align with previous research on γδ NKT cells in *H. sapiens* [89,90,107], which suggests that CD1D-restricted γδ T cells may serve as a bridge between innate and adaptive immunity. By broadening the antigen scope recognized by the TR through SHM and the high diversity of the TRD rearrangements with multiple TRDD genes, camel γδ T cells may have adapted to a range of pathogens in their arid environments [111]. Additionally, this study underscores the potential for the camelid immune system to provide models for understanding *H. sapiens* γδ T cell interactions with non-peptide antigens as well as the evolutionary drivers behind γδ T cell expansion and diversity in γδ high species.

## 5. Conclusions

Our study provides new insights into the complex immunological landscape of camels, suggesting that CD1D-restricted γδ T cells may represent a functional analog to human γδ NKT cells. Our findings can be positioned within the broader context of protein–protein interaction modeling and highlight how protein variants and CDR length can modify affinity and specificity toward targets such as MHC and CD1D. Our computational framework facilitates a deeper understanding of how CDR3 length and variability may impact immune responses, suggesting possible avenues for future research into the mechanistic underpinnings of T cell recognition. Future studies could investigate the in vivo role of these cells in pathogen defense and lipid antigen recognition, as well as their potential as targets for immunotherapeutic applications based on cross-species immune functionality.

## Figures and Tables

**Table 1:** Protein–protein (Pr-Pr) interactions in the *Camelus dromedarius* (Camdro) TRG_TRD in complex with Camdro CD1D. (**A**). Protein–protein interactions between the Camdro TRG V-GAMMA (clone RTS88) and TRD V-DELTA (clone SC19) domains. (**B**). Protein–protein interactions between the Camdro TR gamma-delta domains in complex with the Camdro CD1D G-ALPHA1-LIKE and G-ALPHA2-LIKE domains. Protein–protein interactions are shown according to the four types, as in Figure 2 (top left); ionic interactions within six Å (solid red-brown line), side chain–side chain hydrogen bonds (dashed red-brown dashes), aromatic–aromatic interactions within 4.4 and 7 Å (solid blue line), and cation–π interactions (F/Y/W…K/R) (dashed blue line). The rearranged TRG V-J and TRD V-(D)-J genes encoding the V-gamma and C-delta domains are shown with the CDR-IMGT lengths (CDR1-IMGT, CDR2-IMGT, and CDR3-IMGT) between brackets and separated by dots [1]: RTS88 V-GAMMA Camdro TRGV1-TRGJ1-1 [6.8.15] and SC19 V-DELTA Camdro TRDV1-TRDD1-D2-D4-D5-TRDJ4 [7.3.37]. IMGT labels are in capital letters [1]. Amino acids (AA) are shown in the three-letter and one-letter abbreviation in parentheses and refer to their standardized description based on their physicochemical properties [40]. CDR-IMGT AA of the V-GAMMA domain are in pale orange cells, whereas CDR-IMGT AA of the V-DELTA domain are in pale blue cells. FR-IMGT AA are in uncolored cells. In B, amino acids of the Camdro CD1D G-ALPHA1-LIKE and G-ALPHA2-LIKE domains (central column in green) in contact with the AA of the Camdro TRG V-GAMMA (left columns) domain are in orange cells or Camdro TRD V-DELTA domain (right columns) are in blue cells.

A. Protein–protein interactions between the Camdro TRG V-GAMMA (clone RTS88) and TRD V-DELTA (clone SC19) domains.									
Camdro TRG					Camdro TRD				
RTS88 V-GAMMA TRGV1-TRGJ1-1 [6.8.15]				Pr-Pr	SC19 V-DELTA TRDV1-D1-D2-D4-D5-TRDJ4 [7.3.37]				
CDR1-IMGT	ASN (N)	37		- - - - -	ASP (D)		111.9	CDR3-IMGT	
CDR1-IMGT	TYR (Y)	38		- - - - -	ARG (R)		111.7	CDR3-IMGT	
FR2-IMGT	HIS (H)	40		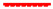	ASP (D)		111.9	CDR3-IMGT	
	HIS (H)	40		- - - - -	ARG (R)		112	CDR3-IMGT	
FR2-IMGT	TYR (Y)	42		- - - - -	ARG (R)		112		
	TYR (Y)	42		- - - - -	ARG (R)		112		
FR2-IMGT	PHE (F)	44		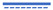	PHE (F)		103	FR3-IMGT	
FR2-IMGT	ARG (R)	52		- - - - -	TRP (W)		111.10	CDR3-IMGT	
FR2-IMGT	TYR (Y)	55		- - - - -	ARG (R)		111.7	CDR3-IMGT	
	TYR (Y)	55		- - - - -	ARG (R)		111.7		
CDR3-IMGT	TYR (Y)	111.1		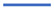	PHE (F)		52	FR2-IMGT	
CDR3-IMGT	SER (S)	112		- - - - -	TRP (W)		112.6	CDR3-IMGT	
CDR3-IMGT	TRP (W)	114		- - - - -	ARG (R)		111.4	CDR3-IMGT	
CDR3-IMGT	ARG (R)	115		- - - - -	PHE (F)		52	FR2-IMGT	
	ARG (R)	115		- - - - -	ASP (D)		107	CDR3-IMT	
CDR3-IMGT	LYS (K)	116		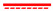	ASP (D)		107	CDR3	
	LYS (K)	116		- - - - -	ASP (D)		107	CDR3	
	LYS (K)	116		- - - - -	TYR (Y)		111.2	CDR3-IMGT	
B. Protein–protein interactions between the Camdro V-gamma_V-delta domains in complex with the Camdro CD1D G-ALPHA-1-LIKE and G-ALPHA-2-LIKE domains.									
Camdro TRG V-GAMMA				Camdro CD1D			Camdro TRD V-DELTA		
RTS88 TRGV1-TRGJ1-1 [6.8.15]							SC19 TRDV1-TRDD1-D2-D4-D5-TRDJ4 [7.3.37]		
V-REGION	AA	IMGT	Pr-Pr	G-ALPHA1-LIKE		Pr-Pr	AA	IMGT	V-REGION
				PHE (F)	55	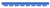	TRP (W)	29	CDR1-IMGT
				GLU (E)	61	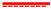	ARG (R)	111.7	CDR3-IMGT
				ASN (N)	62	- - - - -	ARG (R)	111.7	CDR3-IMGT
				HIS (H)	65	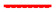	ASP (D)	111.9	CDR3-IMGT
CDR1-IMGT	TYR (Y)	38	- - - - -	HIS (H)	65				
CDR2-IMGT	ASP (D)	57	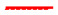	HIS (H)	65				
CDR2-IMGT	LYS (K)	64	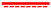	ASP (D)	43				
				G-ALPHA2-LIKE					
				SER (S)	72A	- - - - -	SER (S)	30	CDR1-IMGT
				TRP (W)	69	- - - - -	ARG (R)	109	CDR3-IMGT
				TRP (W)	69	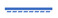	TRP (W)	111.5	CDR3-IMGT
				GLU (E)	65	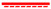	ARG (R)	55	FR2-IMGT
				GLU (E)	65	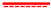	ARG (R)	111.4	CDR3-IMGT
				GLU (E)	65	- - - - -	ARG (R)	111.4	CDR3-IMGT
CDR3-IMGT	TRP (W)	114	- - - - -	GLU (E)	65				

## Data Availability

The data supporting the findings of this study are available from the corresponding authors upon reasonable request.

## Supplementary Materials

The following supporting information can be downloaded at: https://www.mdpi.com/article/10.3390/antib14020046/s1, Figure S1: T cell receptor (TR)/lipid antigen-CD1D complex [1,12,13,36,50]; Figure S2: CDR3-IMGT nucleotide (nt) and predicted amino acid (AA) sequences retrieved from the *Camelus dromedarius* TRD cDNA clones isolated from blood [44]; Figure S3: CDR3-IMGT nucleotide (nt) and predicted amino acid (AA) sequences retrieved from the *Camelus dromedarius* TRD cDNA clones isolated from tonsils [44]; Figure S4: Sequence-structure alignment of the investigated *C. dromedarius* TRG (**A**) and TRD (**B**) chains with human TRG and TRD chains from the crystallized structures 4lhu.pdb [51] and 1hxm.pdb [112]. The sequence-structure alignment of the human (CD1D) (**C**) (from 4lhu.pdb) and B2M (**D**) (from 4lhu.pdb) with their closest homologues in *C. dromedarius*, is also reported. Figure S5: Structural Assessment of the generated 3D protein complex models; Table S1: Interaction energies estimated at the protein interface between TR gamma and TR delta chains or between the reported TR gamma/delta and CD1D; Table S2: List of the all detected interactions within the *C. dromedarius* protein TR gamma (clone RTS88)/delta (clone SC19) in complex with the camel antigen-presenting glycoprotein CD1D and B2M; Table S3: Correspondence between the IMGT DOMAIN numbering, the IMGT file numbering, the PDB numbering for the residues (amino acid 3-letter and one-letter abbreviations) of the domains 1. VGAMMA, 2. V-DELTA, 3. G-ALPHA1-LIKE and G-ALPHA2-LIKE of the PDB entry 4lh4 (IMGT/3Dstructure-DB card for 4lh4, https://www.imgt.org/3Dstructure-DB/cgi/details.cgi?pdbcode=4LHU, accessed on 4 March 2025, IMGT numbering comparison).

## References

[1] Lefranc M.-P. (2014). Immunoglobulin and T Cell Receptor Genes: IMGT^®^ and the Birth and Rise of Immunoinformatics. Front. Immunol..

[2] Lefranc M.-P., Lefranc G. (2001). The Immunoglobulin Factsbook.

[3] Lefranc M.-P., Lefranc G. (2020). Immunoglobulins or Antibodies: IMGT^®^ Bridging Genes, Structures and Functions. Biomedicines.

[4] Lefranc M.-P., Lefranc G. (2001). The T Cell Receptor FactsBook.

[5] Adams E.J., Gu S., Luoma A.M. (2015). Human Gamma Delta T Cells: Evolution and Ligand Recognition. Cell. Immunol..

[6] Van Rhijn I., Le Nours J. (2021). CD1 and MR1 Recognition by Human γδ T Cells. Mol. Immunol..

[7] Lefranc M.-P. (2000). Nomenclature of the Human Immunoglobulin Genes. Curr. Protoc. Immunol..

[8] Lefranc M.-P. (2000). Nomenclature of the Human T Cell Receptor Genes. Curr. Protoc. Immunol..

[9] Lefranc M.-P. (2007). WHO-IUIS Nomenclature Subcommittee for Immunoglobulins and T Cell Receptors Report. Immunogenetics.

[10] Lefranc M.-P. (2008). WHO-IUIS Nomenclature Subcommittee for Immunoglobulins and T Cell Receptors Report August 2007, 13th International Congress of Immunology, Rio de Janeiro, Brazil. Dev. Comp. Immunol..

[11] Stamatopoulos K., Bruford E., Campo E., Lefranc M.-P. (2024). Immunogenetics in Hematopathology and Hematology: Why a Common Language Is Important. Leukemia.

[12] Lefranc M.-P., Pommié C., Ruiz M., Giudicelli V., Foulquier E., Truong L., Thouvenin-Contet V., Lefranc G. (2003). IMGT Unique Numbering for Immunoglobulin and T Cell Receptor Variable Domains and Ig Superfamily V-like Domains. Dev. Comp. Immunol..

[13] Lefranc M.-P., Pommié C., Kaas Q., Duprat E., Bosc N., Guiraudou D., Jean C., Ruiz M., Da Piédade I., Rouard M. (2005). IMGT Unique Numbering for Immunoglobulin and T Cell Receptor Constant Domains and Ig Superfamily C-like Domains. Dev. Comp. Immunol..

[14] Lefranc M.-P., Duprat E., Kaas Q., Tranne M., Thiriot A., Lefranc G. (2005). IMGT Unique Numbering for MHC Groove G-DOMAIN and MHC Superfamily (MhcSF) G-LIKE-DOMAIN. Dev. Comp. Immunol..

[15] Wu D., Yin R., Chen G., Ribeiro-Filho H.V., Cheung M., Robbins P.F., Mariuzza R.A., Pierce B.G. (2024). Structural Characterization and AlphaFold Modeling of Human T Cell Receptor Recognition of NRAS Cancer Neoantigens. bioRxiv.

[16] Saotome K., Dudgeon D., Colotti K., Moore M.J., Jones J., Zhou Y., Rafique A., Yancopoulos G.D., Murphy A.J., Lin J.C. (2023). Structural Analysis of Cancer-Relevant TCR-CD3 and Peptide-MHC Complexes by CryoEM. Nat. Commun..

[17] Dong D., Zheng L., Lin J., Zhang B., Zhu Y., Li N., Xie S., Wang Y., Gao N., Huang Z. (2019). Structural Basis of Assembly of the Human T Cell Receptor–CD3 Complex. Nature.

[18] Rödström K.E.J., Regenthal P., Bahl C., Ford A., Baker D., Lindkvist-Petersson K. (2016). Two Common Structural Motifs for TCR Recognition by Staphylococcal Enterotoxins. Sci. Rep..

[19] Chan K.F., Gully B.S., Gras S., Beringer D.X., Kjer-Nielsen L., Cebon J., McCluskey J., Chen W., Rossjohn J. (2018). Divergent T-Cell Receptor Recognition Modes of a HLA-I Restricted Extended Tumour-Associated Peptide. Nat. Commun..

[20] Le Nours J., Praveena T., Pellicci D.G., Gherardin N.A., Ross F.J., Lim R.T., Besra G.S., Keshipeddy S., Richardson S.K., Howell A.R. (2016). Atypical Natural Killer T-Cell Receptor Recognition of CD1d-Lipid Antigens. Nat. Commun..

[21] Birkinshaw R.W., Pellicci D.G., Cheng T.Y., Keller A.N., Sandoval-Romero M., Gras S., De Jong A., Uldrich A.P., Moody D.B., Godfrey D.I. (2015). αβ T Cell Antigen Receptor Recognition of CD1a Presenting Self Lipid Ligands. Nat. Immunol..

[22] Rödström K.E.J., Regenthal P., Lindkvist-Petersson K. (2015). Structure of Staphylococcal Enterotoxin E in Complex with TCR Defines the Role of TCR Loop Positioning in Superantigen Recognition. PLoS ONE.

[23] Roy S., Ly D., Li N.S., Altman J.D., Piccirilli J.A., Moody D.B., Adams E.J. (2014). Molecular Basis of Mycobacterial Lipid Antigen Presentation by CD1c and Its Recognition by αβ T Cells. Proc. Natl. Acad. Sci. USA.

[24] Van Rhijn I., Kasmar A., De Jong A., Gras S., Bhati M., Doorenspleet M.E., De Vries N., Godfrey D.I., Altman J.D., De Jager W. (2013). A Conserved Human T Cell Population Targets Mycobacterial Antigens Presented by CD1b. Nat. Immunol..

[25] Broughton S.E., Petersen J., Theodossis A., Scally S.W., Loh K.L., Thompson A., van Bergen J., Kooy-Winkelaar Y., Henderson K.N., Beddoe T. (2012). Biased T Cell Receptor Usage Directed against Human Leukocyte Antigen DQ8-Restricted Gliadin Peptides Is Associated with Celiac Disease. Immunity.

[26] Deng L., Langley R.J., Brown P.H., Xu G., Teng L., Wang Q., Gonzales M.I., Callender G.G., Nishimura M.I., Topalian S.L. (2007). Structural Basis for the Recognition of Mutant Self by a Tumor-Specific, MHC Class II-Restricted T Cell Receptor. Nat. Immunol..

[27] Kjer-Nielsen L., Borg N.A., Pellicci D.G., Beddoe T., Kostenko L., Clements C.S., Williamson N.A., Smyth M.J., Besra G.S., Reid H.H. (2006). A Structural Basis for Selection and Cross-Species Reactivity of the Semi-Invariant NKT Cell Receptor in CD1d/Glycolipid Recognition. J. Exp. Med..

[28] Gadola S.D., Koch M., Marles-Wright J., Lissin N.M., Shepherd D., Matulis G., Harlos K., Villiger P.M., Stuart D.I., Jakobsen B.K. (2006). Structure and Binding Kinetics of Three Different Human CD1d-α-Galactosylceramide-Specific T Cell Receptors. J. Exp. Med..

[29] Chen J.L., Stewart-Jones G., Bossi G., Lissin N.M., Wooldridge L., Choi E.M.L., Held G., Dunbar P.R., Esnouf R.M., Sami M. (2005). Structural and Kinetic Basis for Heightened Immunogenicity of T Cell Vaccines. J. Exp. Med..

[30] Kjer-Nielsen L., Clements C.S., Brooks A.G., Purcell A.W., McCluskey J., Rossjohn J. (2002). The 1.5 Å Crystal Structure of a Highly Selected Antiviral T Cell Receptor Provides Evidence for a Structural Basis of Immunodominance. Structure.

[31] Hoque M., Grigg J.B., Ramlall T., Jones J., McGoldrick L.L., Lin J.C., Olson W.C., Smith E., Franklin M.C., Zhang T. (2025). Structural Characterization of Two γδ TCR/CD3 Complexes. Nat. Commun..

[32] Wegrecki M., Ocampo T.A., Gunasinghe S.D., von Borstel A., Tin S.Y., Reijneveld J.F., Cao T.P., Gully B.S., Le Nours J., Moody D.B. (2022). Atypical Sideways Recognition of CD1a by Autoreactive γδ T Cell Receptors. Nat. Commun..

[33] Allison T.J., Winter C.C., Fournié J.J., Bonneville M., Garboczi D.N. (2001). Structure of a Human γδ T-Cell Antigen Receptor. Nature.

[34] Xin W., Huang B., Chi X., Liu Y., Xu M., Zhang Y., Li X., Su Q., Zhou Q. (2024). Structures of Human γδ T Cell Receptor–CD3 Complex. Nature.

[35] Kaas Q., Ruiz M., Lefranc M.-P. (2004). IMGT/3Dstructure-DB and IMGT/StructuralQuery, a Database and a Tool for Immunoglobulin, T Cell Receptor and MHC Structural Data. Nucleic Acids Res..

[36] Ehrenmann F., Lefranc M.-P. (2011). Imgt/3Dstructure-DB: Querying the IMGT Database for 3D Structures in Immunology and Immunoinformatics (IG or Antibodies, TR, MH, RPI, and FPIA). Cold Spring Harb. Protoc..

[37] Lefranc M.-P., Lefranc G. (2022). IMGT/3Dstructure-DB: T-Cell Receptor TR Paratope and Peptide/Major Histocompatibility pMH Contact Sites and Epitope. Methods Mol. Biol..

[38] Ehrenmann F., Kaas Q., Lefranc M.-P. (2009). IMGT/3Dstructure-DB and IMGT/DomainGapAlign: A Database and a Tool for Immunoglobulins or Antibodies, T Cell Receptors, MHC, IgSF and MHcSF. Nucleic Acids Res..

[39] Ehrenmann F., Lefranc M.-P. (2011). IMGT/DomainGapAlign: IMGT Standardized Analysis of Amino Acid Sequences of Variable, Constant, and Groove Domains (IG, TR, MH, IgSF, MhSF). Cold Spring Harb. Protoc..

[40] Pommié C., Levadoux S., Sabatier R., Lefranc G., Lefranc M.-P. (2004). IMGT Standardized Criteria for Statistical Analysis of Immunoglobulin V-Region Amino Acid Properties. J. Mol. Recognit..

[41] Ehrenmann F., Giudicelli V., Duroux P., Lefranc M.-P. (2011). IMGT/Collier de Perles: IMGT Standardized Representation of Domains (IG, TR, and IgSF Variable and Constant Domains, MH and MhSF Groove Domains). Cold Spring Harb. Protoc..

[42] Lefranc M.-P., Lefranc G. (2024). Using IMGT Unique Numbering for IG Allotypes and Fc-Engineered Variants of Effector Properties and Half-Life of Therapeutic Antibodies. Immunol. Rev..

[43] Antonacci R., Massari S., Linguiti G., Jambrenghi A.C., Giannico F., Lefranc M.-P., Ciccarese S. (2020). Evolution of the T-Cell Receptor (TR) Loci in the Adaptive Immune Response: The Tale of the TRG Locus in Mammals. Genes.

[44] Antonacci R., Mineccia M., Lefranc M.-P., Ashmaoui H.M.E., Lanave C., Piccinni B., Pesole G., Hassanane M.S., Massari S., Ciccarese S. (2011). Expression and Genomic Analyses of *Camelus dromedarius* T Cell Receptor Delta (TRD) Genes Reveal a Variable Domain Repertoire Enlargement Due to CDR3 Diversification and Somatic Mutation. Mol. Immunol..

[45] Vaccarelli G., Antonacci R., Tasco G., Yang F., Giordano L., El Ashmaoui H.M., Hassanane M.S., Massari S., Casadio R., Ciccarese S. (2012). Generation of Diversity by Somatic Mutation in the *Camelus dromedarius* T-Cell Receptor Gamma Variable Domains. Eur. J. Immunol..

[46] Ciccarese S., Vaccarelli G., Lefranc M.-P., Tasco G., Consiglio A., Casadio R., Linguiti G., Antonacci R. (2014). Characteristics of the Somatic Hypermutation in the *Camelus dromedarius* T Cell Receptor Gamma (TRG) and Delta (TRD) Variable Domains. Dev. Comp. Immunol..

[47] Ciccarese S., Burger P.A., Ciani E., Castelli V., Linguiti G., Plasil M., Massari S., Horin P., Antonacci R. (2019). The Camel Adaptive Immune Receptors Repertoire as a Singular Example of Structural and Functional Genomics. Front. Genet..

[48] Massari S., Linguiti G., Giannico F., D’addabbo P., Ciccarese S., Antonacci R. (2021). The Genomic Organisation of the Tra/Trd Locus Validates the Peculiar Characteristics of Dromedary δ-Chain Expression. Genes.

[49] Hussein B.A., Hallner A., Wennström L., Brune M., Martner A., Hellstrand K., Bernson E., Thorén F.B. (2021). Impact of NK Cell Activating Receptor Gene Variants on Receptor Expression and Outcome of Immunotherapy in Acute Myeloid Leukemia. Front. Immunol..

[50] Linguiti G., Tragni V., Pierri C.L., Massari S., Lefranc M.-P., Antonacci R., Ciccarese S. (2022). 3D Structures Inferred from CDNA Clones Identify the CD1D-Restricted γδ T Cell Receptor in Dromedaries. Front. Immunol..

[51] Uldrich A.P., Le Nours J., Pellicci D.G., Gherardin N.A., Mcpherson K.G., Lim R.T., Patel O., Beddoe T., Gras S., Rossjohn J. (2013). CD1d-Lipid Antigen Recognition by the γδ TCR. Nat. Immunol..

[52] Lobley A., Sadowski M.I., Jones D.T. (2009). PGenTHREADER and PDomTHREADER: New Methods for Improved Protein Fold Recognition and Superfamily Discrimination. Bioinformatics.

[53] Zhang Y. (2008). I-TASSER Server for Protein 3D Structure Prediction. BMC Bioinform..

[54] Trisolini L., Gambacorta N., Gorgoglione R., Montaruli M., Laera L., Colella F., Volpicella M., De Grassi A., Pierri C.L. (2019). FAD/NADH Dependent Oxidoreductases: From Different Amino Acid Sequences to Similar Protein Shapes for Playing an Ancient Function. J. Clin. Med..

[55] Pierri C.L., Parisi G., Porcelli V. (2010). Computational Approaches for Protein Function Prediction: A Combined Strategy from Multiple Sequence Alignment to Molecular Docking-Based Virtual Screening. Biochim. Biophys. Acta (BBA)—Proteins Proteom..

[56] Mercurio I., Tragni V., Busto F., De Grassi A., Pierri C.L. (2020). Protein Structure Analysis of the Interactions between SARS-CoV-2 Spike Protein and the Human ACE2 Receptor: From Conformational Changes to Novel Neutralizing Antibodies. Cell. Mol. Life Sci..

[57] Pierri C.L., Bossis F., Punzi G., De Grassi A., Cetrone M., Parisi G., Tricarico D. (2016). Molecular Modeling of Antibodies for the Treatment of TNFα-Related Immunological Diseases. Pharmacol. Res. Perspect..

[58] Krieger E., Joo K., Lee J., Lee J., Raman S., Thompson J., Tyka M., Baker D., Karplus K. (2009). Improving Physical Realism, Stereochemistry, and Side-Chain Accuracy in Homology Modeling: Four Approaches That Performed Well in CASP8. Proteins Struct. Funct. Bioinform..

[59] Patskovsky Y., Natarajan A., Patskovska L., Nyovanie S., Joshi B., Morin B., Brittsan C., Huber O., Gordon S., Michelet X. (2023). Author Correction: Molecular Mechanism of Phosphopeptide Neoantigen Immunogenicity. Nat. Commun..

[60] Guan Y., Chen J., Guan H., Chen T.-T., Teng Y., Wei Z., Li Z., Ouyang S., Chen X. (2024). Structural and Functional Characterization of a Fish Type I Subgroup d IFN Reveals Its Binding to Receptors. J. Immunol..

[61] Slater B.T., Han X., Chen L., Xiong Y. (2020). Structural Insight into T Cell Coinhibition by PD-1H (VISTA). Proc. Natl. Acad. Sci. USA.

[62] Bovay A., Zoete V., Rizkallah P.J., Beck K., Delbreil P., Speiser D.E., Cole D.K., Fuertes Marraco S.A. (2020). Identification of a Superagonist Variant of the Immunodominant Yellow Fever Virus Epitope NS4b 214-222 by Combinatorial Peptide Library Screening. Mol. Immunol..

[63] Sibener L.V., Fernandes R.A., Kolawole E.M., Carbone C.B., Liu F., McAffee D., Birnbaum M.E., Yang X., Su L.F., Yu W. (2018). Isolation of a Structural Mechanism for Uncoupling T Cell Receptor Signaling from Peptide-MHC Binding. Cell.

[64] Lehrer S., Rheinstein P.H. (2020). Alignment of Alzheimer’s Disease Amyloid-β Peptide and Herpes Simplex Virus-1 PUL15 C-Terminal Nuclease Domain. J. Alzheimer’s Dis. Rep..

[65] Tragni V., Mercurio I., Paoletti D.P., Onofrio A., Laera L., Cafferati Beltrame L., Sgobba M.N., Guerra L., Volpicella M., De Grassi A. (2023). Deconstructing SARS-CoV-2 Neutralization: A Modular Molecular Framework for Computational Design and Comparison of Antibodies and Nanobodies Targeting the Spike RBD. J. Med. Virol..

[66] Rodriguez J.A., Gonzalez J., Arboleda-Bustos C.E., Mendoza N., Martinez C., Pinzon A. (2022). Computational Modeling of the Effect of Five Mutations on the Structure of the ACE2 Receptor and Their Correlation with Infectivity and Virulence of Some Emerged Variants of SARS-CoV-2 Suggests Mechanisms of Binding Affinity Dysregulation. Chem. Biol. Interact..

[67] Goswami A., Kumar M., Ullah S., Gore M.M. (2023). De Novo Design of Anti-Variant COVID-19 Vaccine. Biol. Methods Protoc..

[68] Sung K.H., Josewski J., Dübel S., Blankenfeldt W., Rau U. (2018). Structural Insights into Antigen Recognition of an Anti-β-(1,6)-β-(1,3)-D-Glucan Antibody. Sci. Rep..

[69] Ilmiawan L., Tjandrawinata R.R., Prasasty V.D. (2024). In Silico Study Binding Affinity of Regdanvimab-RBD Spike Protein SARS CoV-2 Omicron Variant Indonesia. J. Appl. Pharm. Sci..

[70] Simister P.C., Border E.C., Vieira J.F., Pumphrey N.J. (2022). Structural Insights into Engineering a T-Cell Receptor Targeting MAGE-A10 with Higher Affinity and Specificity for Cancer Immunotherapy. J. Immunother. Cancer.

[71] Van Durme J., Delgado J., Stricher F., Serrano L., Schymkowitz J., Rousseau F. (2011). A Graphical Interface for the FoldX Forcefield. Bioinformatics.

[72] Schymkowitz J., Borg J., Stricher F., Nys R., Rousseau F., Serrano L. (2005). The FoldX Web Server: An Online Force Field. Nucleic Acids Res..

[73] Lanzarotti E., Marcatili P., Nielsen M. (2018). Identification of the Cognate Peptide-MHC Target of T Cell Receptors Using Molecular Modeling and Force Field Scoring. Mol. Immunol..

[74] Abdelfattah N.S., Kula T., Elledge S.J. (2024). T-Switch: A Specificity-Based Engineering Platform for Developing Safe and Effective T Cell Therapeutics. Immunity.

[75] Schaap-Johansen A.L., Vujović M., Borch A., Hadrup S.R., Marcatili P. (2021). T Cell Epitope Prediction and Its Application to Immunotherapy. Front. Immunol..

[76] van der Kant R., Karow-Zwick A.R., Van Durme J., Blech M., Gallardo R., Seeliger D., Aßfalg K., Baatsen P., Compernolle G., Gils A. (2017). Prediction and Reduction of the Aggregation of Monoclonal Antibodies. J. Mol. Biol..

[77] Buß O., Rudat J., Ochsenreither K. (2018). FoldX as Protein Engineering Tool: Better Than Random Based Approaches?. Comput. Struct. Biotechnol. J..

[78] Rosace A., Bennett A., Oeller M., Mortensen M.M., Sakhnini L., Lorenzen N., Poulsen C., Sormanni P. (2023). Automated Optimisation of Solubility and Conformational Stability of Antibodies and Proteins. Nat. Commun..

[79] Sanchez R., Sali A., Sánchez R., Sali A. (2000). Comparative Protein Structure Modeling. Introduction and Practical Examples with Modeller. Methods Mol. Biol..

[80] Martí-Renom M.A., Stuart A.C., Fiser A., Sánchez R., Melo F., Šali A. (2000). Comparative Protein Structure Modeling of Genes and Genomes. Annu. Rev. Biophys. Biomol. Struct..

[81] Waterhouse A.M., Procter J.B., Martin D.M.A., Clamp M., Barton G.J. (2009). Jalview Version 2-A Multiple Sequence Alignment Editor and Analysis Workbench. Bioinformatics.

[82] Linguiti G., Kossida S., Pierri C.L., Jabado-Michaloud J., Folch G., Massari S., Lefranc M.-P., Ciccarese S., Antonacci R. (2021). The t Cell Receptor (Trb) Locus in Tursiops Truncatus: From Sequence to Structure of the Alpha/Beta Heterodimer in the Human/Dolphin Comparison. Genes.

[83] Yang J., Zhang Y. (2015). Protein Structure and Function Prediction Using I-TASSER. Curr. Protoc. Bioinform..

[84] Studer G., Rempfer C., Waterhouse A.M., Gumienny R., Haas J., Schwede T. (2020). QMEANDisCo—Distance Constraints Applied on Model Quality Estimation. Bioinformatics.

[85] Kryshtafovych A., Schwede T., Topf M., Fidelis K., Moult J. (2023). Critical Assessment of Methods of Protein Structure Prediction (CASP)—Round XV. Proteins Struct. Funct. Bioinform..

[86] Williams C.J., Headd J.J., Moriarty N.W., Prisant M.G., Videau L.L., Deis L.N., Verma V., Keedy D.A., Hintze B.J., Chen V.B. (2018). MolProbity: More and Better Reference Data for Improved All-Atom Structure Validation. Protein Sci..

[87] Croll T.I., Sammito M.D., Kryshtafovych A., Read R.J. (2019). Evaluation of Template-Based Modeling in CASP13. Proteins Struct. Funct. Bioinform..

[88] Lüthy R., Bowie J.U., Eisenberg D. (1992). Assessment of Protein Models with Three-Dimensional Profiles. Nature.

[89] Cheng T.-Y., Praveena T., Govindarajan S., Almeida C.F., Pellicci D.G., Arkins W.C., Van Rhijn I., Venken K., Elewaut D., Godfrey D.I. (2024). Lipidomic Scanning of Self-Lipids Identifies Headless Antigens for Natural Killer T Cells. Proc. Natl. Acad. Sci. USA.

[90] Matsuo Y., Tsujimura T., Drexler H.G. (2021). Proposal for the Designation of the Natural Killer Antigens-Positive γδ T-Cell Subset as γδ NKT-Cells: Nomenclature Based on Immunoprofile. Hum. Cell.

[91] Mallevaey T., Clarke A.J., Scott-Browne J.P., Young M.H., Roisman L.C., Pellicci D.G., Patel O., Vivian J.P., Matsuda J.L., McCluskey J. (2011). A Molecular Basis for NKT Cell Recognition of CD1d-Self-Antigen. Immunity.

[92] Macho-Fernandez E., Brigl M. (2015). The Extended Family of CD1d-Restricted NKT Cells: Sifting through a Mixed Bag of TCRs, Antigens, and Functions. Front. Immunol..

[93] Wun K.S., Ross F., Patel O., Besra G.S., Porcelli S.A., Richardson S.K., Keshipeddy S., Howell A.R., Godfrey D.I., Rossjohn J. (2012). Human and Mouse Type I Natural Killer T Cell Antigen Receptors Exhibit Different Fine Specificities for CD1d-Antigen Complex. J. Biol. Chem..

[94] Pellicci D.G., Uldrich A.P., Le Nours J., Ross F., Chabrol E., Eckle S.B.G., de Boer R., Lim R.T., McPherson K., Besra G. (2014). The Molecular Bases of δ/αβ T Cell-Mediated Antigen Recognition. J. Exp. Med..

[95] Marrack P., Scott-Browne J.P., Dai S., Gapin L., Kappler J.W. (2008). Evolutionarily Conserved Amino Acids That Control TCR-MHC Interaction. Annu. Rev. Immunol..

[96] Ekeruche-Makinde J., Miles J.J., Van Den Berg H.A., Skowera A., Cole D.K., Dolton G., Schauenburg A.J.A., Tan M.P., Pentier J.M., Llewellyn-Lacey S. (2013). Peptide Length Determines the Outcome of TCR/Peptide-MHCI Engagement. Blood.

[97] Cao T.P., Shahine A., Cox L.R., Besra G.S., Moody D.B., Rossjohn J. (2024). A Structural Perspective of How T Cell Receptors Recognize the CD1 Family of Lipid Antigen–Presenting Molecules. J. Biol. Chem..

[98] Ling Sok C., Rossjohn J., Gully B.S. (2024). The Evolving Portrait of Gd TCR Recognition Determinants. J. Immunol..

[99] Taylor W.R. (2020). Exploring Protein Fold Space. Biomolecules.

[100] Gromiha M.M., Selvaraj S. (2004). Inter-Residue Interactions in Protein Folding and Stability. Prog. Biophys. Mol. Biol..

[101] Zhou H.X., Pang X. (2018). Electrostatic Interactions in Protein Structure, Folding, Binding, and Condensation. Chem. Rev..

[102] Rossjohn J., Pellicci D.G., Patel O., Gapin L., Godfrey D.I. (2012). Recognition of CD1d-Restricted Antigens by Natural Killer T Cells. Nat. Rev. Immunol..

[103] Modi T., Campitelli P., Kazan I.C., Ozkan S.B. (2021). Protein Folding Stability and Binding Interactions through the Lens of Evolution: A Dynamical Perspective. Curr. Opin. Struct. Biol..

[104] Scheiblhofer S., Laimer J., Machado Y., Weiss R., Thalhamer J. (2017). Influence of Protein Fold Stability on Immunogenicity and Its Implications for Vaccine Design. Expert. Rev. Vaccines.

[105] Deseke M., Prinz I. (2020). Ligand Recognition by the γδ TCR and Discrimination between Homeostasis and Stress Conditions. Cell. Mol. Immunol..

[106] Zhu Y., Zhang W., Veerapen N., Besra G., Cresswell P. (2010). Calreticulin Controls the Rate of Assembly of CD1d Molecules in the Endoplasmic Reticulum. J. Biol. Chem..

[107] Girardi E., Zajonc D.M. (2012). Molecular Basis of Lipid Antigen Presentation by CD1d and Recognition by Natural Killer T Cells. Immunol. Rev..

[108] DeWitt W.S., Yu K.K.Q., Wilburn D.B., Sherwood A., Vignali M., Day C.L., Scriba T.J., Robins H.S., Swanson W.J., Emerson R.O. (2018). A Diverse Lipid Antigen–Specific TCR Repertoire Is Clonally Expanded during Active Tuberculosis. J. Immunol..

[109] De Libero G., Mori L. (2006). How T Lymphocytes Recognize Lipid Antigens. FEBS Lett..

[110] Pierce B.G., Vreven T., Weng Z. (2014). Modeling T Cell Receptor Recognition of CD1-Lipid and MR1-Metabolite Complexes. BMC Bioinform..

[111] Zhao Y., Lin L., Xiao Z., Li M., Wu X., Li W., Li X., Zhao Q., Wu Y., Zhang H. (2018). Protective Role of γδ T Cells in Different Pathogen Infections and Its Potential Clinical Application. J. Immunol. Res..

[112] Xu B., Pizarro J.C., Holmes M.A., McBeth C., Groh V., Spies T., Strong R.K. (2011). Crystal structure of a γδ T-cell receptor specific for the human MHC class I homolog MICA. Proc. Natl. Acad. Sci..

