# Supplementary material for: Three-Dimensional Modeling of Camelus dromedarius T Cell Receptor Gamma (TRG)_Delta (TRD)/CD1D Complex Reveals Different Binding Interactions Depending on the TRD CDR3 Length
Source: Antibodies (Basel). 2025 May 29;14(2):46. doi: 10.3390/antib14020046 (PMC12189835; doi:10.3390/antib14020046)
Supplement: Supplementary file 1 [file antibodies-14-00046-s001.zip › antibodies-3511851-supplementary/Suppl.Mat.Fig.Tab/Figure S2 blood.jpg.OK.pdf]

ACAGTATACGGAC	GGTGGGATTTC	ACTGGCTGGACTC	GGTGGGATTTC	ACGATACGTGGAC	GCCAGGAGAC
TRDD1	TRDD2	TRDD4	TRDD*	TRDD5	TRDD6

CLONES	SUBGROUPs	CDR3	TRDD	CDR3 length (AA)
b11	TRDV1	GCTCTAaaaaagtggggctgggtgggattcataggatacgtggtaactgggacggggCCACTGATAT<br>A L K K W G W V G F I G Y V V T G T G P L I	D2_D*_D5	22
b13	TRDV1	GCTCTCAGAGAAGcttgggaatggtggagACTGATAT<br>A L R E A W E W W R L I	D2_D*	12
b14	TRDV1	GCTCTTGATGgggggagaggtatacgggtgggactggctggtgggatttttggggctgggttacGATCCACTGATAT<br>A L D G G E R V Y G W D W L V G F L G L G Y D P L I	D1_D2_D4_D*	26
b16	TRDV1	GCTCTTGccccacccttacagtatgggcctgtactggttggaccgggggccccgctggaggtctacgatgctgggTGATAT<br>A L A P P L Q Y G P V L V G P G G P A G G L R C W V I	ND	27
b18	TRDV1	GCTCcctttattctggtgggatgggtgacggtgggTCCACTGATAT<br>A P F I L V G W V T V G P L I	D2_D*	15
b19	TRDV1	GCTCcgacgacggacgtggggtactggctggacggattttaagtggacgacgtgtggacatgggggTCCACTGATAT<br>A P D D G R G V L A G R I L S G R R V D M G G P L I	D2_D4_D5	26
b110	TRDV1	GCTCTTGATGAtagggggggccttatacgaggctgggaccacggGGATCCACTGATAT<br>A L D D R G G L I R G W D H G D P L I	D1_D2	19
b111	TRDV1	GCTCTTGATGAAAacaagagtatacggggccctactggagggtgggaccgagggtggccctcgccTTGAAAGCACAGCTTGTC<br>A L D E N K S I R G P Y W R V G P R V A L A L K A Q L V	D1_D2_D*	28
b112	TRDV1	GCgttcccgtatacggggtgggtttgggatttctcTCCACTGATAT<br>A F P Y T G W G L G F S P L I	D1_D2_D*	15
b113	TRDV1	GCTCTCagtagaagatatgggggcttagggCCACTGATAT<br>A L S R R Y G G L G P L I	D1_D4	13
b114	TRDV1	GCTCcgcctcgggggctggactcgatgggatcccttacgatacaaccgggagacggCCACTGATAT<br>A P P R G A G L D G I P Y D T T G R R P L I	D4	22
b115	TRDV1	GCTCTCTGGGAccgcccgttcggagacgacgtgcccagttATCCACTGATAT<br>A L W D R P F G D D V P S Y P L I	D6	17
b119	TRDV1	GCTCTCTGGGAAAagtatgggtgggttacgtggagggagactcggCCACTGATAT<br>A L W E K Y G W V T W R E T R P L I	D1_D2_D5_D6	18
b120	TRDV1	GCTCcccttcgcactggatggagagactgcgGAAAGCACAGCTTATCT<br>A P P S H W M E R L R K A Q L I	ND	16
b121	TRDV1	GCTCTGTGGGtcaaaattcagagtatacggacgtggggtttcggtggacccccgtggaccgcgaggggaggaaCTACTAAAGGCAAGTTCGTC<br>A L W V K I Q S I R T W G F G G P R G P R G G G T T K G K F V	D1_D2_D*_D5	31
b122	TRDV1	GCTCTCAGAGAAGactggggtttcgctggacgggcgctgggagaccccGGGACACCCGACAGATGTTTT<br>A L R E D W G F A G R A L G D P R D T R Q M F	D2_D6	23
b123	TRDV1	GCTCTCAGAGAggggtcgaggctcggtgggagtggggagacggtCCTCTGATAT<br>A L R E G V E A R W E W G D G P L I	D2_D*	18
b124	TRDV1	GCTCTCAGAGccctaggggaggggttgactcgaggatacgtgggaGACCCACTGATAT<br>A L R A L G E G L T R G Y V G D P L I	D2_D5	19
b125	TRDV1	GCTCTCAGAGAgagtatactggggtactggctggtgggattgggagAGGATCCACTGATAT<br>A L R E S I L G Y W L V G L G E D P L I	D1_D4_D*	20
b126	TRDV1	GCTCTCAGAGAtctttgggtatacggacgaggggtttactccaAGCTTATC<br>A L R D L W V Y G R G V Y S K L I	D1	17
b128	TRDV1	GCTCTCAGAGggaattgggtaacggctggacgggatggacggatacgtgggagagtagaggCACTGATAT<br>A L R G N W V T A G R D G R I R G R V E A L I	D4_D5	23
b129	TRDV1	GCTCTCTGGGAAAtcgtcacggacgggctgtggtcccgatacgtggaggacggGGATCCGCTGATTT<br>A L W E I V T D G L W S R Y V E D G D P L I	D5	22
D4b12	TRDV3	GTTCTGGAGTACggggtggatctcattacgatacgtgggagactGGATCCACTGATAT<br>V L E Y G V D L I T I R G R L D P L I	D5	19
D4b110	TRDV3	GTTCTGGAGggtttgggatttcagacgacacgtgggagaCAGGATCCACTGATAT<br>V L E G L G F Q T T R G R Q D P L I	D2_D5	18

TR delta blood cDNA clones
