# Supplementary material for: Three-Dimensional Modeling of Camelus dromedarius T Cell Receptor Gamma (TRG)_Delta (TRD)/CD1D Complex Reveals Different Binding Interactions Depending on the TRD CDR3 Length
Source: Antibodies (Basel). 2025 May 29;14(2):46. doi: 10.3390/antib14020046 (PMC12189835; doi:10.3390/antib14020046)
Supplement: Supplementary file 1 [file antibodies-14-00046-s001.zip › antibodies-3511851-supplementary/Suppl.Mat.Fig.Tab/Figure S3.jpg.OK.pdf]

ACAGTATACGGAC TRDD1 | GGTGGGATTTC TRDD2 | ACTGGCTGGACTC TRDD4 | GGTGGGATTTC TRDD* | ACGATACGTGGAC TRDD5 | GCCAGGAGAC TRDD6

CLONES	SUBGROUPs	CDR3	TRDD	CDR3 length (AA)
ts6	TRDV1	GCTCTCTGGGAgctagtacttcgtggttcggggggagacacacaaT<br>A L W E L V L R G S G G D T Q	ND	15
ts11	TRDV1	GCTCTCTGGGAccgcccgttcggagacgacgtgcccagttacCCACTGATAT<br>A L W D R P F G D D V P S Y P L I	ND	17
ts19	TRDV1	GCTCTCTGcgaaagaagggtgtggggt**actggctggactc**ggggatcccaacgaggcgtgaggagacctcctcTATCTACTAAAGGCAAGTTCGTCT<br>A L C E R R V W G **T G W T** R G S Q R G V R R P P L S T K G K F V	D2_D4_D5_D6	32
ts30	TRDV1	gtgttcccgcatacggggtggggttt**gggattt**tctCCACTGATAT<br>V F P H T G W G L **G F** S P L I	D1_D2_D*	15
ts31	TRDV1	GCTCTTGATGAAAacaagagtatacggggccctactggagggtgggaccgag**ggtgg**ccctcgccTTGAAAGCACAGCTTATCT<br>A L D E N K S I R G P Y W R V G P R **V** A L A L K A Q L I	D1_D2_D*	28
ts32	TRDV1	GCTCTAaaaaagtggggctg**ggtgggatt**cataggatacgtggtaactgggacggggCCACTGATAT<br>A L K K W G W **V G F** I G Y V V T G T G P L I	D2_D*_D5	22
ts58	TRDV1	GCgttcccgtatacggggtggggttt**gggattt**tctCCACTGATAT<br>A F P Y T G W G L **G F** S P L I	D1_D2_D*	15
ts62	TRDV1	GCTCTCagagatgggaaggctgggggtggggtgggattcccattc**gcctggac**ggtgcgggagacgggTCCACTGATAT<br>A L R D G K A G G G V G F P F **A W T** V R E T G P L I	D2_D4_D6	26
ts9	TRAV33/DV6	GCTCTCTGGGATTACgtgggggggg**ggtggga**tcggcccgatacgtggagggagactcCCACTGATA<br>A L W D Y V G G G G I G P I R G G R L P L I	D2_D*_D5	22
ts11	TRAV33/DV6	GCTCTCTGGCGaccatacggcggc**gctggactc**gggggggctgcACTGATAT<br>A L W R P Y G G **A G L** G G A A L I	D1_D4	17
ts13	TRAV33/DV6	GCTCTCTGGCGcccatacggtgggatc**ggact**aggggggAGCACAGCTTATCT<br>A L W R P Y G G I **G L** G G A Q L I	D1_D2_D4	17
ts21	TRAV33/DV6	GCTCTCTGGcgtccgcacggttcagtatccctccggcacgtgggagacttGGATCCACTGATAT<br>A L W R P H G S V S L R H V G D L D P L I	D1_D6	21
ts1	TRDV3	GTcggcccccctcgtgggacggggtacgataggccgggagactATCCACTGATAT<br>V G P P R G T G Y D R P G D Y P L I	D2_D6	18
ts5	TRDV3	GTTCcgggaggtggacagctACTGATAT<br>V P G G G Q L L I	D2	9
ts7	TRDV3	GTTCTGGgcacaacagtgggagccggcggTCCACTGATAT<br>V L G T T V G A G G P L I	D2	13

TR delta tonsils cDNA clones
