# Supplementary material for: Three-Dimensional Modeling of Camelus dromedarius T Cell Receptor Gamma (TRG)_Delta (TRD)/CD1D Complex Reveals Different Binding Interactions Depending on the TRD CDR3 Length
Source: Antibodies (Basel). 2025 May 29;14(2):46. doi: 10.3390/antib14020046 (PMC12189835; doi:10.3390/antib14020046)
Supplement: Supplementary file 1 [file antibodies-14-00046-s001.zip › antibodies-3511851-supplementary/Suppl.Mat.Fig.Tab/Figure.S5.StructureAssessment.pdf]

RTS88.SC19.CD1D.b-microglobulin

## Structure Assessment

RTS88g.SC19.CD1D.b.pdb *Created: Wed, Apr 9, 13:08*

PROJECT DATA ⌄ Compare ☐

Ramachandran Plots

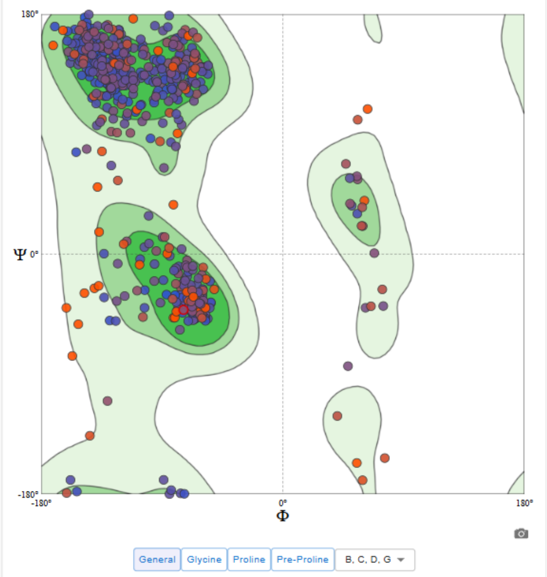


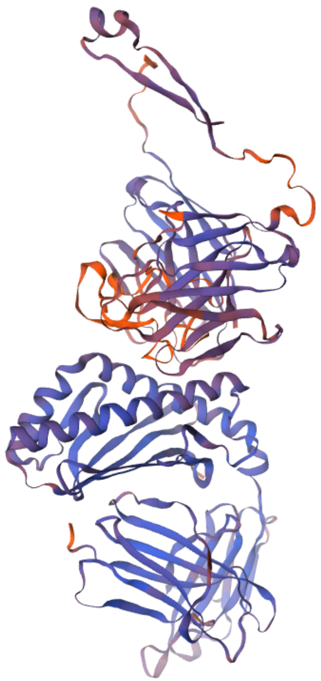

## QMEANDisCo

QMEANDisCo Global: 0.73 ±0.05

Coordinates with QMEAN local scores in the B-factor column

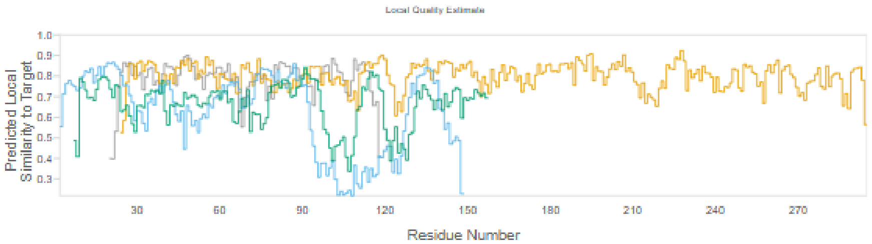


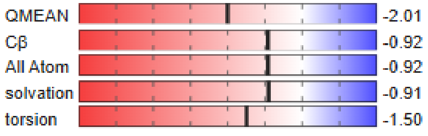


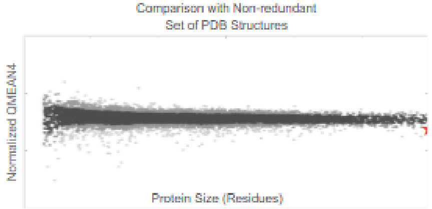


## MolProbity Results

MolProbity Score 2.27

☐ Clash Score 4.70

(G132 PRO-G132 PRO), (G45 ARG-G60 ASP), (D52 ASP-D54 SER), (C78 ASP-C79 GLN), (B76 SER-B77 LYS), (D123 ARG-G38 TYR), (C152 ASP-C155 SER)

Ramachandran Favoured 93.65%

☐ Ramachandran Outliers 1.21%

D125 ASN, G105 SER, D111 LYS, G112 GLY, D52 ASP, D99 VAL, D104 GLY, C271 ALA

☐ Rotamer Outliers 6.24%

D33 TYR, B90 THR, C197 SER, D131 LYS, G122 LEU, C182 SER, B106 LEU, B29 VAL, B21 ILE, G40 ASP, C98 ASP, D141 LYS, C170 ARG, B88 ASN, D71 GLU, C221 LEU, D122 SER, B83 LEU, G155 LEU, D95 GLU, D117 TRP, D69 LYS, G10 SER, D125 ASN, D37 PHE, D92 SER, D39 GLN, D139 PRO, D99 VAL, D12 SER, B108 GLU, D109 THR, G111 PHE, D106 THR, C87 ILE, G138 LEU, C114 ILE

☐ C-Beta Deviations 6

G105 SER, D99 VAL, G98 TRP, C87 ILE, G36 TYR, D125 ASN

☐ Bad Bonds 23 / 5544

G132 PRO, D147 PRO, C153 PRO, C217 PRO, G139 PRO, C246 PRO, G36 TYR, D9 PRO, C230 TYR, C262 TYR, C151 PRO, B40 PRO, G134 PRO, C111 PRO, D41 PRO, B34 PRO, B51 HIS, G34 HIS, D14 HIS, D82 HIS, B33 HIS, G36 TYR-G37 LYS, C156 PRO

☐ Bad Angles 35 / 7533

(B67 LYS-B68 ALA), B67 LYS, G106 GLY, C153 PRO, D147 PRO, D139 PRO, C157 PRO, G107 TRP, (G106 GLY-G107 TRP), D104 GLY, D98 THR, G98 TRP, D14 HIS, D82 HIS, D33 TYR, G36 TYR, D125 ASN, C217 PRO, B33 HIS, B51 HIS, C213 PRO, G34 HIS, D101 GLY, D113 ALA, D95 GLU, (G107 TRP-G108 ARG), D110 GLN, C110 TYR, D94 ASP, C246 PRO, C241 GLY

☐ Cis Non-Proline 1 / 630

(B67 LYS-B68 ALA)

☐ Cis Prolines 1 / 35

(B51 HIS-B52 PRO)

☐ Twisted Non-Proline 3 / 630

(D51 GLN-D52 ASP), (D96 ARG-D97 SER), (D123 ARG-D124 VAL)

*Results obtained using MolProbity version 4.4*

# RTS88.SC44.CD1D.b-microglobulin

## Structure Assessment

RTS88g.SC44.CD1D.b.pdb *Created: Wed, Apr 9, 13:09*

PROJECT DATA

Compare

Ramachandran Plots

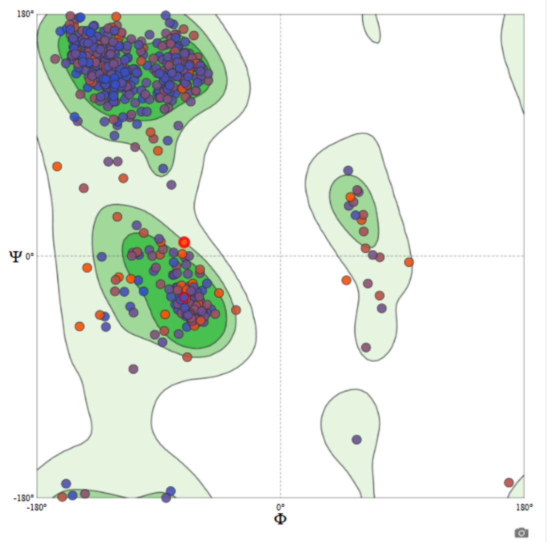


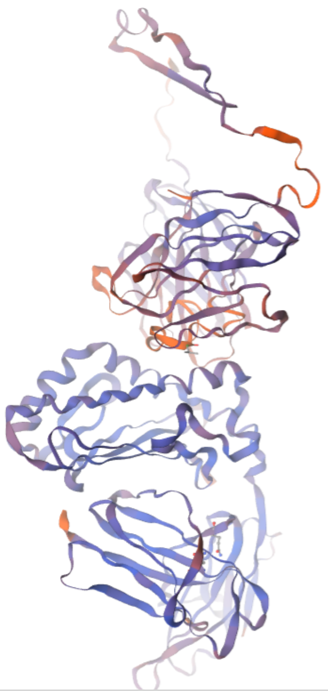

## QMEANDisCo

QMEANDisCo Global: 0.73 ±0.05

Coordinates with QMEAN local scores in the B-factor column

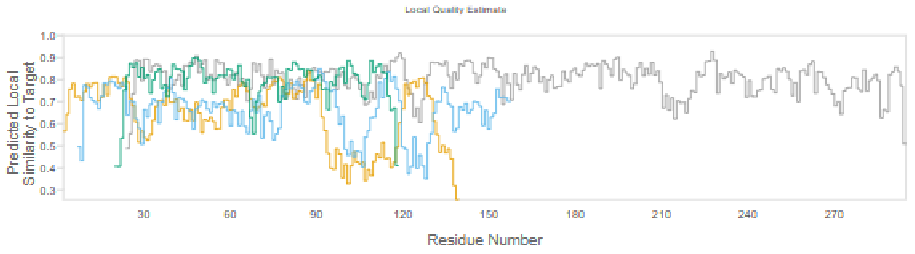


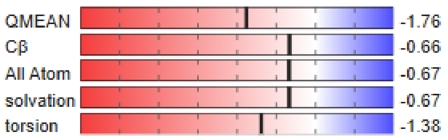


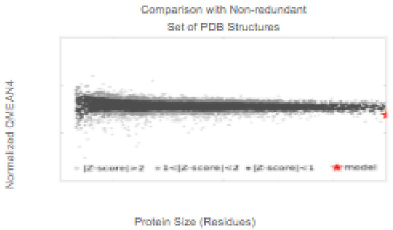


## MolProbity Results

| | |
|---|---|
| MolProbity Score | 2.21 |
| Clash Score | 4.61 |
| (G132 PRO-G132 PRO), (D138 PRO-D138 PRO), (B76 SER-B77 LYS), (B118 GLN-C225 HIS), (B44 ASN-C256 GLN), (B46 TYR-C255 PRO) | |
| Ramachandran Favoured | 94.94% |
| Ramachandran Outliers | 0.77% |
| G102 LYS, D97 GLY, G108 ARG, D117 GLN, D114 ILE | |
| Rotamer Outliers | 6.63% |
| B90 THR, C64 THR, C82 GLU, D117 GLN, D19 VAL, B106 LEU, B29 VAL, B21 ILE, C143 SER, D81 SER, D53 ASP, C150 VAL, C66 ARG, B88 ASN, G41 ARG, D96 LEU, C201 LYS, C39 SER, C141 VAL, G155 LEU, G117 LEU, B83 LEU, C65 ILE, G122 LEU, C211 SER, C111 PRO, G30 VAL, C217 PRO, C123 LEU, C209 LEU, G49 TYR, C204 GLN, B108 GLU, D74 ILE, C114 ILE, C152 ASP, G14 LYS, G101 ARG | |
| C-Beta Deviations | 0 |
| Bad Bonds | 19 / 5409 |
| D138 PRO, G132 PRO, D41 PRO, G139 PRO, G134 PRO, C246 PRO, D83 PRO, C230 TYR, C157 PRO, C262 TYR, C233 PRO, B40 PRO, B34 PRO, C202 PRO, B51 HIS, G34 HIS, B33 HIS, C153 PRO, D130 PRO | |
| Bad Angles | 23 / 7341 |
| (B67 LYS-B68 ALA), (D101 ARG-D102 ASP), D101 ARG, B67 LYS, D99 TYR, D138 PRO, (D115 GLY-D116 ALA), C217 PRO, G34 HIS, C213 PRO, C111 PRO, C110 TYR, D117 GLN, (D116 ALA-D117 GLN), C241 GLY, B33 HIS, B51 HIS, D130 PRO | |
| Cis Non-Proline | 3 / 622 |
| (D101 ARG-D102 ASP), (D115 GLY-D116 ALA), (B67 LYS-B68 ALA) | |
| Cis Prolines | 1 / 34 |
| (B51 HIS-B52 PRO) | |
| Twisted Non-Proline | 4 / 622 |
| (D33 THR-D34 LEU), (D98 VAL-D99 TYR), (G100 LEU-G101 ARG), (G107 TRP-G108 ARG) | |

*Results obtained using MolProbity version 4.4*

RTS88.SC54.CD1D.b-microglobulin

Structure Assessment

RTS88g.SC54.CD1D.b.pdb *Created: Wed, Apr 9, 13:09*

PROJECT DATA

Compare

Ramachandran Plots

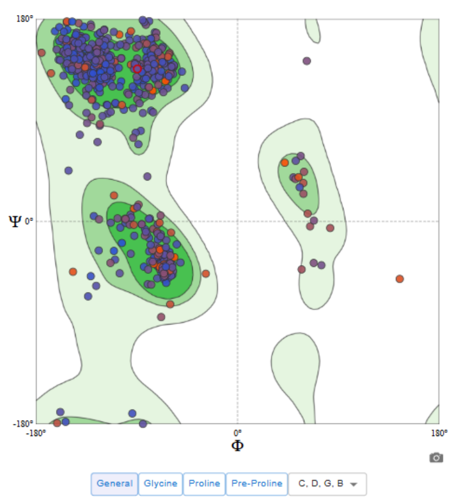


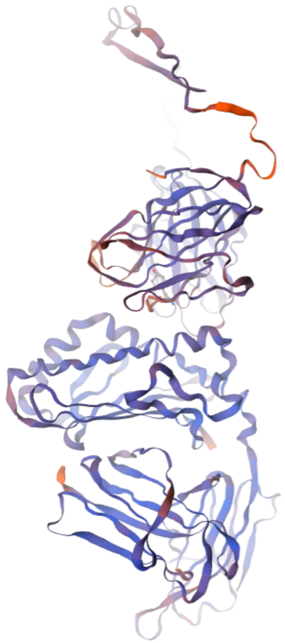

## MolProbity Results

| | |
|---|---|
| MolProbity Score | 2.15 |
| Clash Score | 5.38 |
| (G132 PRO-G132 PRO), (C217 PRO-C217 PRO), (B117 ASP-C211 SER), (B76 SER-B77 LYS), (B118 GLN-C264 ARG) | |
| Ramachandran Favoured | 96.07% |
| Ramachandran Outliers | 0.79% |
| G102 LYS, C271 ALA, D95 SER, D52 TYR, D96 TYR | |
| Rotamer Outliers | 5.96% |
| C219 ARG, B90 THR, C141 VAL, C150 VAL, G64 ASP, C206 GLU, G125 ASP, B106 LEU, B29 VAL, B21 ILE, G144 ILE, G40 ASP, G101 ARG, G12 THR, C258 ASP, B88 ASN, C280 ARG, B83 LEU, C152 ASP, C72 SER, D28 SER, D50 GLN, C181 ASP, D97 SER, G14 LYS, C38 ASN, C61 ASP, C52 GLU, B108 GLU, C114 ILE, D34 ILE, G30 VAL, C64 THR, C129 LYS | |
| C-Beta Deviations | 0 |
| Bad Bonds | 24 / 5328 |
| G132 PRO, C217 PRO, D123 PRO, C151 PRO, C246 PRO, C202 PRO, C233 PRO, G139 PRO, D96 TYR, C230 TYR, C262 TYR, B40 PRO, D9 PRO, B34 PRO, C153 PRO, D41 PRO, B51 HIS, B33 HIS, D95 SER-D96 TYR, D14 HIS, C25 HIS, G147 HIS, G34 HIS, C111 PRO | |
| Bad Angles | 17 / 7235 |
| (B67 LYS-B68 ALA), B67 LYS, C217 PRO, C67 PHE, D115 PRO, D14 HIS, C25 HIS, G34 HIS, B33 HIS, B51 HIS, G147 HIS, (C270 ALA-C271 ALA), C213 PRO, C70 PRO | |
| Cis Non-Proline | 1 / 605 |
| (B67 LYS-B68 ALA) | |
| Cis Prolines | 1 / 35 |
| (B51 HIS-B52 PRO) | |
| Twisted Non-Proline | 4 / 605 |
| (D94 PRO-D95 SER), (D95 SER-D96 TYR), (D96 TYR-D97 SER), (G100 LEU-G101 ARG) | |

*Results obtained using MolProbity version 4.4*

## QMEANDisCo

QMEANDisCo Global: 0.76 ±0.05

Coordinates with QMEAN local scores in the B-factor column

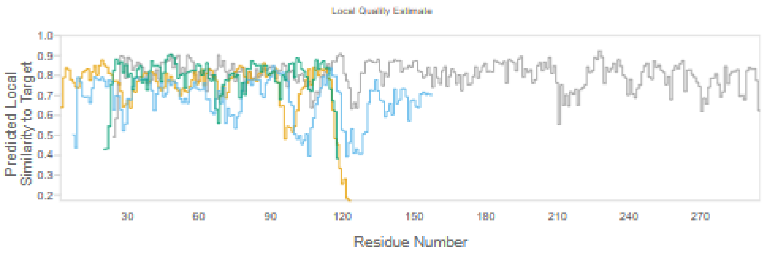


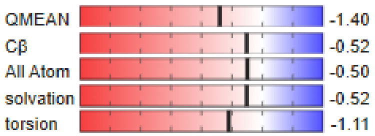


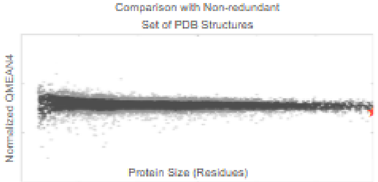

5R1S69.SC19.CD1D.b-microglobulin

## Structure Assessment

5r1s69_SC19_CD1D.b.mg.pdb *Created: Wed, Apr 9, 14:06*

PROJECT DATA

Compare

Ramachandran Plots

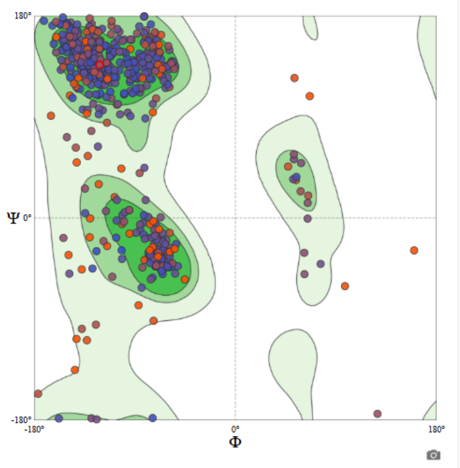


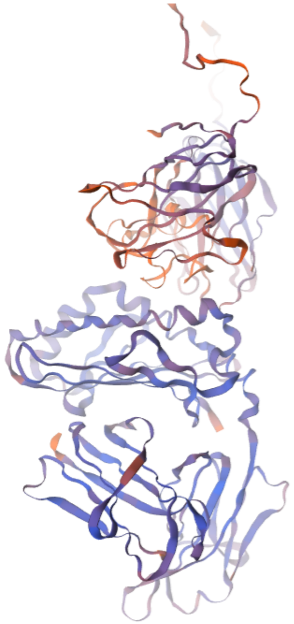

## QMEANDisCo

QMEANDisCo Global: 0.71 ±0.05

Coordinates with QMEAN local scores in the B-factor column

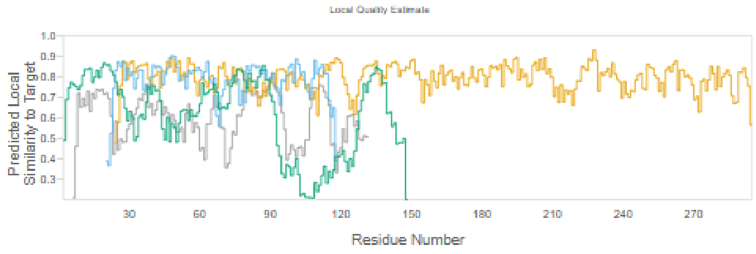


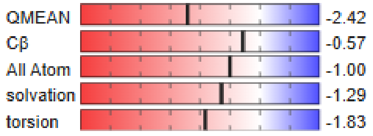


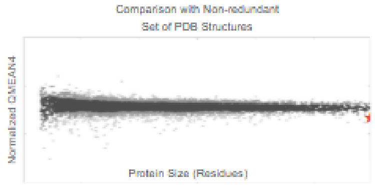


## MolProbity Results

| | |
|---|---|
| MolProbity Score | 2.11 |
| Clash Score<br>(C156 PRO-C156 PRO) | 2.95 |
| Ramachandran Favoured | 93.55% |
| Ramachandran Outliers<br>G96 ALA, D111 LYS, D104 GLY, G73 ASP, G60 VAL, D96 ARG, G99 ALA, D52 ASP | 1.26% |
| Rotamer Outliers<br>C85 GLN, C150 VAL, G47 TYR, G75 ARG, B106 LEU, D146 THR, D117 TRP, C220 LEU, D108 TRP, G76 SER, C141 VAL, D59 LYS, C200 GLU, B83 LEU, B78 ASP, C277 LEU, C39 SER, G52 LYS, C38 ASN, G131 PRO, D30 SER, G64 ASP, D12 SER, D95 GLU, G9 LEU, D139 PRO, G100 PRO, D99 VAL, D111 LYS, D47 PHE, C114 ILE, C69 LYS, D51 GLN, D96 ARG, D105 ARG | 6.23% |
| C-Beta Deviations<br>D99 VAL, D111 LYS, G97 TRP, G60 VAL | 4 |
| Bad Bonds<br>C156 PRO, B52 PRO, B109 PRO, C217 PRO, G126 PRO, D9 PRO, D41 PRO, G124 PRO, C246 PRO, G21 PRO, C230 TYR, C262 TYR, G34 TYR, C111 PRO, C70 PRO, D147 PRO, D14 HIS, B33 HIS, B51 HIS, C86 HIS, G100 PRO, D82 HIS | 22 / 5307 |
| Bad Angles<br>(B67 LYS-B68 ALA), B67 LYS, C217 PRO, G100 PRO, D33 TYR, D101 GLY, D139 PRO, G131 PRO, D110 GLN, G101 ASP, G97 TRP, D103 TRP, (G99 ALA-G100 PRO), C111 PRO, (G100 PRO-G101 ASP), D82 HIS, (D51 GLN-D52 ASP), D14 HIS, G72 ASN, C241 GLY, C86 HIS, (D112 GLY-D113 ALA), G102 ASN, B51 HIS, D96 ARG, D52 ASP, B33 HIS, G103 LYS, B40 PRO, D98 THR, G99 ALA, (D95 GLU-D96 ARG), C157 PRO, D104 GLY, (G59 SER-G60 VAL), C70 PRO, G21 PRO, C67 PHE, C213 PRO, G124 PRO, B86 HIS, C107 HIS, D125 ASN | 51 / 7216 |
| Cis Non-Proline<br>(B67 LYS-B68 ALA) | 1 / 603 |
| Cis Prolines<br>(B51 HIS-B52 PRO) | 1 / 37 |
| Twisted Non-Proline<br>(G96 ALA-G97 TRP), (D50 ARG-D51 GLN), (D96 ARG-D97 SER), (D103 TRP-D104 GLY), (D113 ALA-D114 TYR), (D127 LEU-D128 ILE), (D128 ILE-D129 PHE) | 7 / 603 |
| Twisted Prolines<br>(G99 ALA-G100 PRO) | 1 / 37 |

*Results obtained using MolProbity version 4.4*

RTS88.SC54.CD1D.b-microglobulin

job #205208: RTS88g.SC54.CD1D.b.pdb

# CD1D

VERIFY3D

**VERIFY3D**
84.01% of the residues have averaged 3D-1D score >= 0.1
**Pass**

At least 80% of the amino acids have scored >= 0.1 in the 3D/1D profile.

Chain C | Chain D | Chain G | Chain B

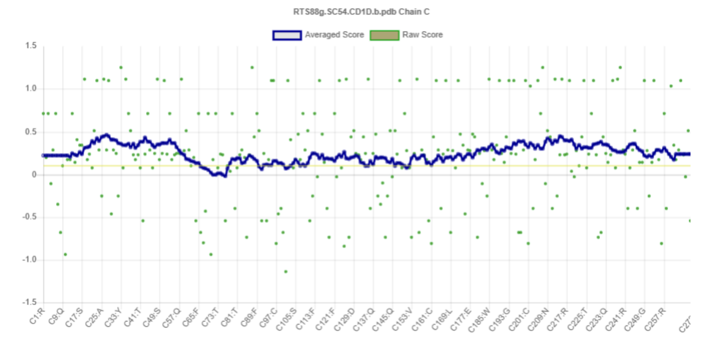


Save as image | Save data to file | DSSP program results

job #205208: RTS88g.SC54.CD1D.b.pdb

# Beta-microglobulin

VERIFY3D

**VERIFY3D**
84.01% of the residues have averaged 3D-1D score >= 0.1
**Pass**

At least 80% of the amino acids have scored >= 0.1 in the 3D/1D profile.

Chain C | Chain D | Chain G | Chain B

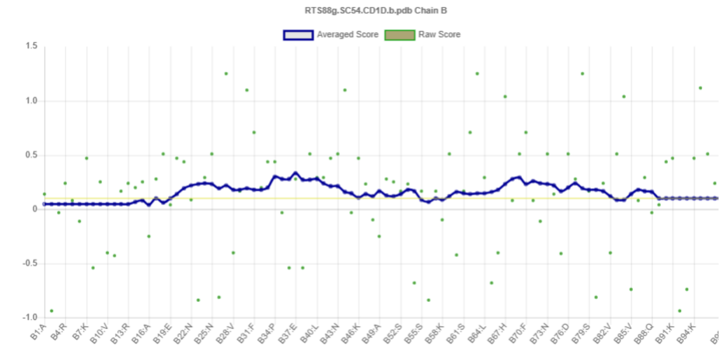


Save as image | Save data to file | DSSP program results

job #205208: RTS88g.SC54.CD1D.b.pdb

# TCRgamma

VERIFY3D

**VERIFY3D**
84.01% of the residues have averaged 3D-1D score >= 0.1
**Pass**

At least 80% of the amino acids have scored >= 0.1 in the 3D/1D profile.

Chain C | Chain D | Chain G | Chain B

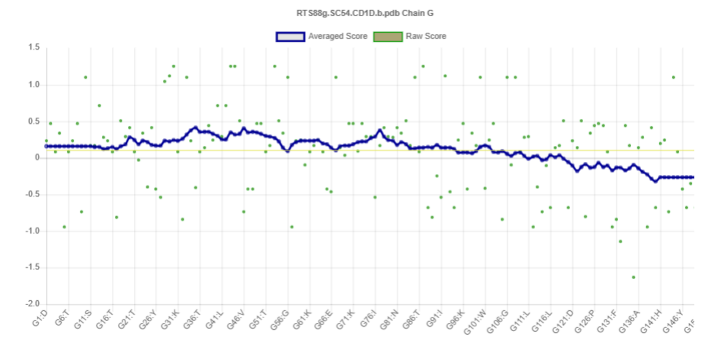


Save as image | Save data to file | DSSP program results

job #205208: RTS88g.SC54.CD1D.b.pdb

# TCRdelta

VERIFY3D

**VERIFY3D**
84.01% of the residues have averaged 3D-1D score >= 0.1
**Pass**

At least 80% of the amino acids have scored >= 0.1 in the 3D/1D profile.

Chain C | Chain D | Chain G | Chain B

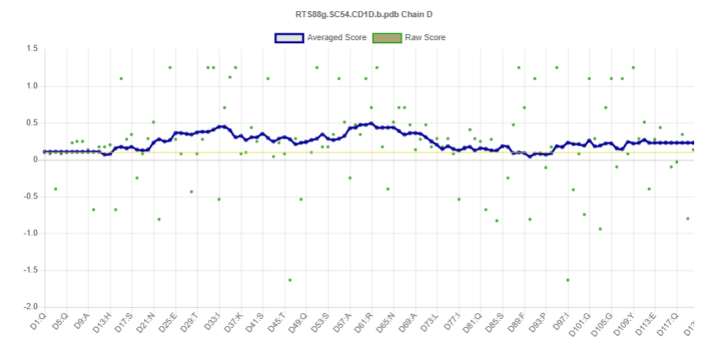


Save as image | Save data to file | DSSP program results

RTS88.SC19.CD1D.b-microglobulin

## CD1D

job #205149: RTS88g.SC19.CD1D.b.pdb

VERIFY3D

**VERIFY3D**
83.86% of the residues have averaged 3D-1D score >= 0.1
**Pass**

At least 80% of the amino acids have scored >= 0.1 in the 3D/1D profile.

Chain B | Chain C | Chain D | Chain G

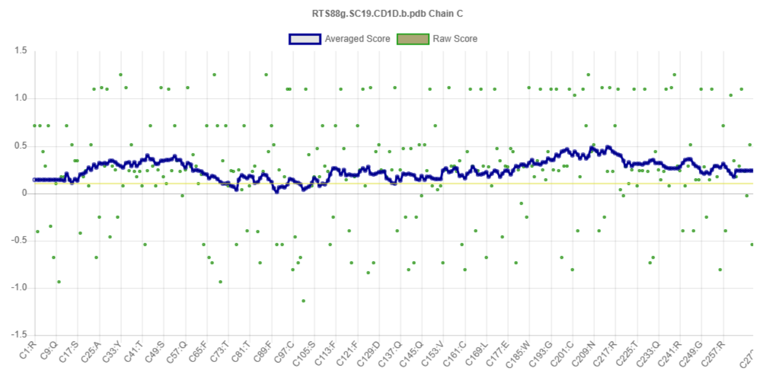


Save as image | Save data to file | DSSP program results

## Beta-microglobulin

job #205149: RTS88g.SC19.CD1D.b.pdb

VERIFY3D

**VERIFY3D**
83.86% of the residues have averaged 3D-1D score >= 0.1
**Pass**

At least 80% of the amino acids have scored >= 0.1 in the 3D/1D profile.

Chain B | Chain C | Chain D | Chain G

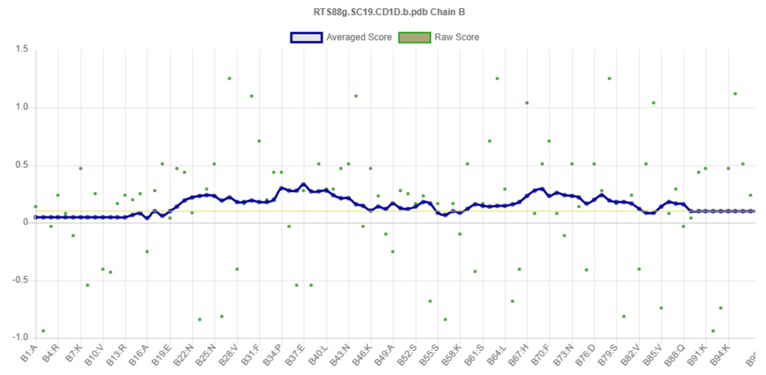


Save as image | Save data to file | DSSP program results

## TCRgamma

job #205149: RTS88g.SC19.CD1D.b.pdb

VERIFY3D

**VERIFY3D**
83.86% of the residues have averaged 3D-1D score >= 0.1
**Pass**

At least 80% of the amino acids have scored >= 0.1 in the 3D/1D profile.

Chain B | Chain C | Chain D | Chain G

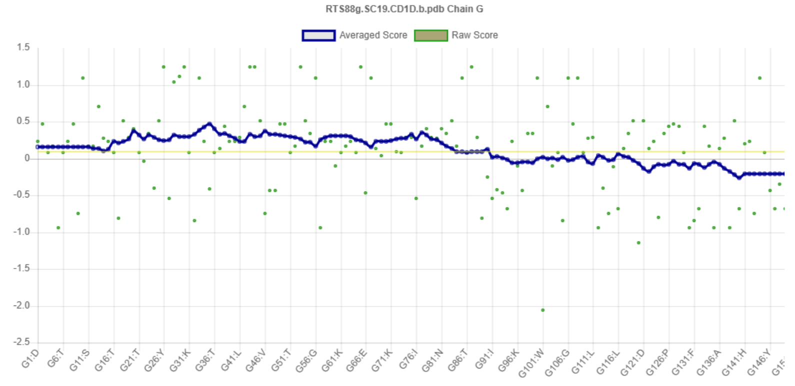


Save as image | Save data to file | DSSP program results

## TCRdelta

job #205149: RTS88g.SC19.CD1D.b.pdb

VERIFY3D

**VERIFY3D**
83.86% of the residues have averaged 3D-1D score >= 0.1
**Pass**

At least 80% of the amino acids have scored >= 0.1 in the 3D/1D profile.

Chain B | Chain C | Chain D | Chain G

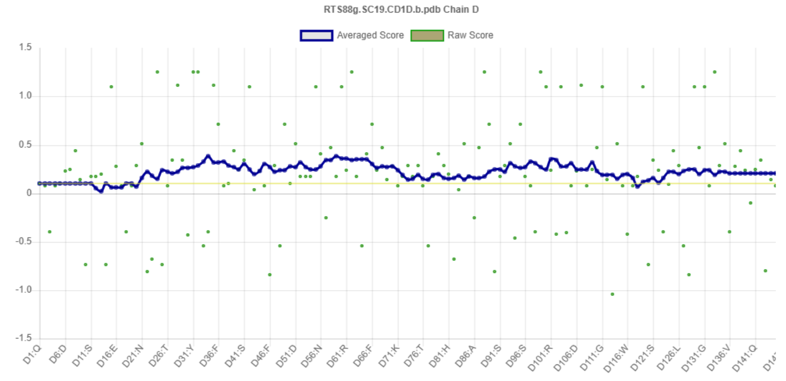


Save as image | Save data to file | DSSP program results

RTS88.SC44.CD1D.b-microglobulin

job #205151: RTS88g.SC44.CD1D.b.pdb

## CD1D

VERIFY3D

VERIFY3D
83.94% of the residues have averaged 3D-1D score >= 0.1
**Pass**

At least 80% of the amino acids have scored >= 0.1 in the 3D/1D profile.

Chain C | Chain D | Chain G | Chain B

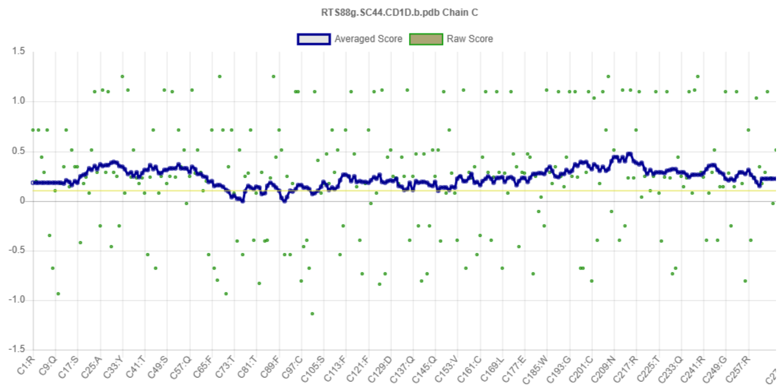


Save as image | Save data to file | DSSP program results

job #205151: RTS88g.SC44.CD1D.b.pdb

## Beta-microglobulin

VERIFY3D

VERIFY3D
83.94% of the residues have averaged 3D-1D score >= 0.1
**Pass**

At least 80% of the amino acids have scored >= 0.1 in the 3D/1D profile.

Chain C | Chain D | Chain G | Chain B

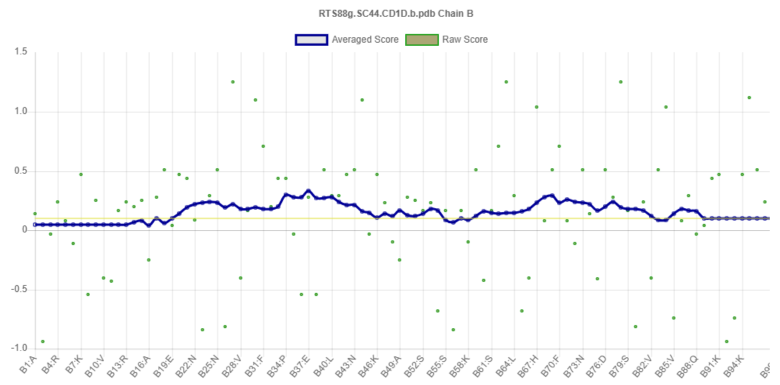


Save as image | Save data to file | DSSP program results

job #205151: RTS88g.SC44.CD1D.b.pdb

## TCRgamma

VERIFY3D

VERIFY3D
83.94% of the residues have averaged 3D-1D score >= 0.1
**Pass**

At least 80% of the amino acids have scored >= 0.1 in the 3D/1D profile.

Chain C | Chain D | Chain G | Chain B

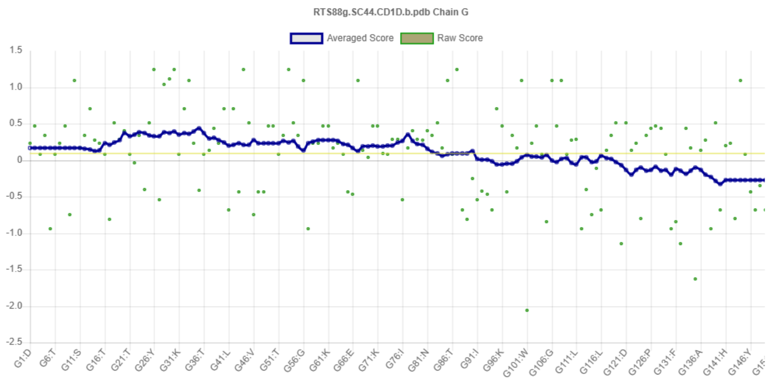


Save as image | Save data to file | DSSP program results

job #205151: RTS88g.SC44.CD1D.b.pdb

## TCRdelta

VERIFY3D

VERIFY3D
83.94% of the residues have averaged 3D-1D score >= 0.1
**Pass**

At least 80% of the amino acids have scored >= 0.1 in the 3D/1D profile.

Chain C | Chain D | Chain G | Chain B

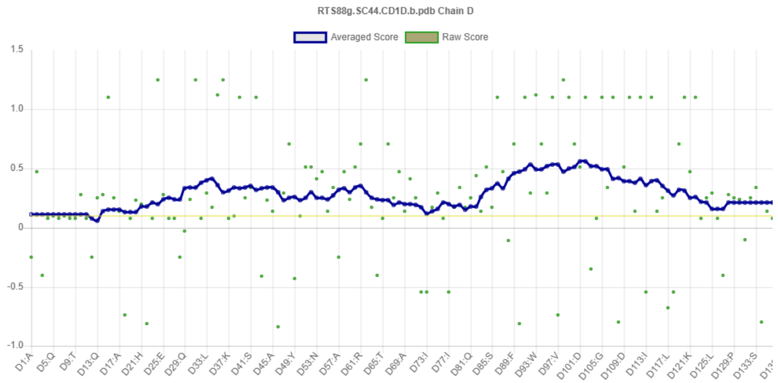


Save as image | Save data to file | DSSP program results

5R1S69.SC19.CD1D.b-microglobulin

## CD1D

job #205210: 5r1s69_SC19_CD1D.b.mg.pdb

VERIFY3D

**VERIFY3D**
90.06% of the residues have averaged 3D-1D score >= 0.1
**Pass**

At least 80% of the amino acids have scored >= 0.1 in the 3D/1D profile.

Chain G | Chain C | Chain B | Chain D

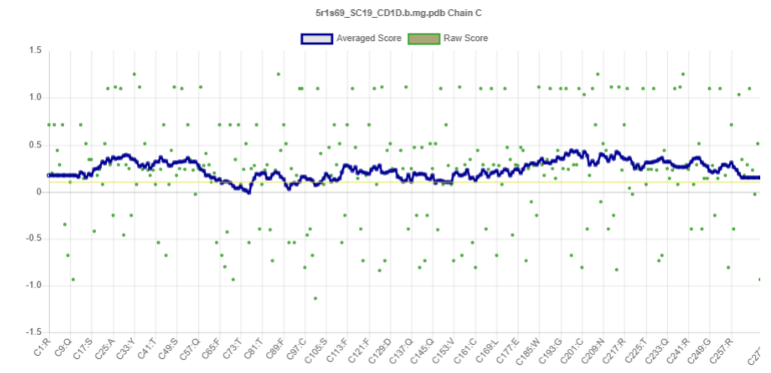


Save as image | Save data to file | DSSP program results

## Beta-microglobulin

job #205210: 5r1s69_SC19_CD1D.b.mg.pdb

VERIFY3D

**VERIFY3D**
90.06% of the residues have averaged 3D-1D score >= 0.1
**Pass**

At least 80% of the amino acids have scored >= 0.1 in the 3D/1D profile.

Chain G | Chain C | Chain B | Chain D

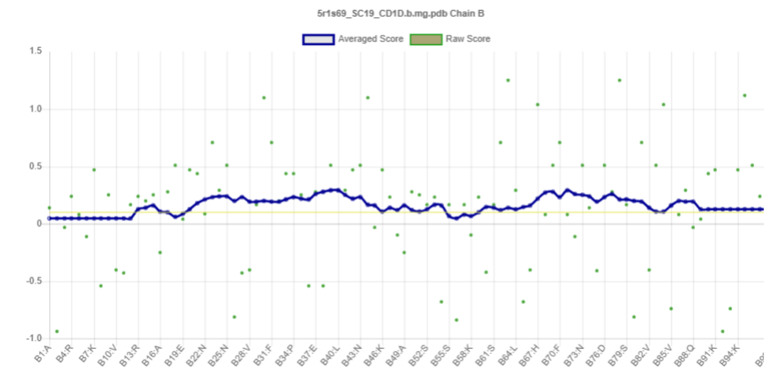


Save as image | Save data to file | DSSP program results

## TCRgamma

job #205210: 5r1s69_SC19_CD1D.b.mg.pdb

VERIFY3D

**VERIFY3D**
90.06% of the residues have averaged 3D-1D score >= 0.1
**Pass**

At least 80% of the amino acids have scored >= 0.1 in the 3D/1D profile.

Chain G | Chain C | Chain B | Chain D

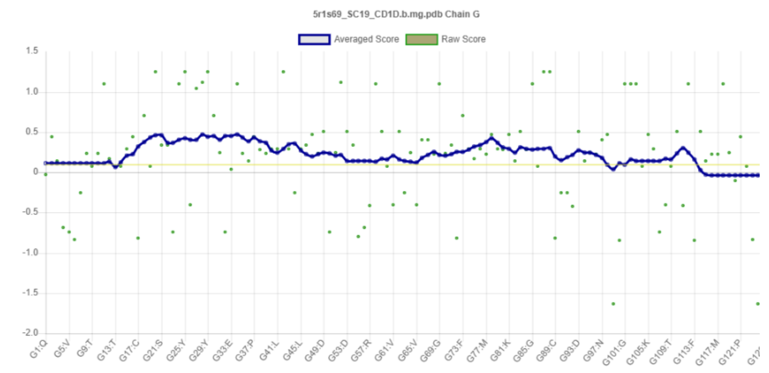


Save as image | Save data to file | DSSP program results

## TCRdelta

job #205210: 5r1s69_SC19_CD1D.b.mg.pdb

VERIFY3D

**VERIFY3D**
90.06% of the residues have averaged 3D-1D score >= 0.1
**Pass**

At least 80% of the amino acids have scored >= 0.1 in the 3D/1D profile.

Chain G | Chain C | Chain B | Chain D

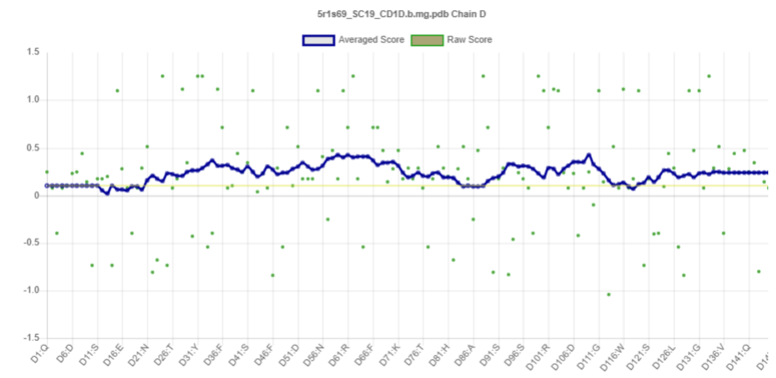


Save as image | Save data to file | DSSP program results
