# Supplementary material for: Three-Dimensional Modeling of Camelus dromedarius T Cell Receptor Gamma (TRG)_Delta (TRD)/CD1D Complex Reveals Different Binding Interactions Depending on the TRD CDR3 Length
Source: Antibodies (Basel). 2025 May 29;14(2):46. doi: 10.3390/antib14020046 (PMC12189835; doi:10.3390/antib14020046)
Supplement: Supplementary file 1 [file antibodies-14-00046-s001.zip › antibodies-3511851-supplementary/Suppl.Mat.Fig.Tab/LegendsToSuppFig.Tables10apr2025.pdf]

3D modeling of *Camelus dromedarius* T cell receptor gamma (TRG)_delta (TRD)/CD1D complex reveals different binding interactions depending on TRD CDR3 length

Salvatrice Ciccarese[a,*] Marie-Paule Lefranc[b], Giulia C.M. Perrone[c], Pietro D'Addabbo[a], Ciro Leonardo Pierri[c,*]

[a] Dipartimento di Bioscienze, Biotecnologie e Ambiente, Università degli Studi di Bari "Aldo Moro", Bari, Italy

[b] IMGT®, the International ImMunoGeneTics Information System® (IMGT), Laboratoire d'ImmunoGénétique Moléculaire (LIGM), Institut de Génétique Humaine (IGH), Centre National de la Recherche Scientifique (CNRS), Université de Montpellier (UM), France

[c] Laboratory of Biochemistry, Structural, and Molecular Biology, Department of Pharmacy - Pharmaceutical Sciences, University of Bari, Via E. Orabona, 4 - 70125 Bari, Italy.

* Correspondence:

Department of Biosciences, Biotechnologies and Environment, University of Bari Aldo Moro, Via E. Orabona, 4- 70125 Bari, Italy. E-mail: salvatricemaria.ciccarese@uniba.it,

Laboratory of Biochemistry, Structural, and Molecular Biology, Department of Pharmacy - Pharmaceutical Sciences, University of Bari, Via E. Orabona, 4 - 70125 Bari, Italy. E-mail: ciro.pierri@uniba.it

Supplementary Figures and Tables legends

Supplementary Tables Legends

Table S1

Interaction energies estimated at the protein interface between TR gamma and TR delta chains or between the reported TR gamma/delta and CD1D. The most significant negative energy values in *Camelus dromedarius* in comparison with the corresponding (4hlu.pdb) ones in *Homo sapiens* are in bold.

Table S2

List of the all detected interactions within the *C. dromedarius* protein TR gamma (clone RTS88)/delta (clone SC19) in complex with the camel antigen-presenting glycoprotein CD1D and B2M. The letter "A" indicates the antigen-presenting glycoprotein CD1D. The letters G and D indicate the camel TR gamma and delta chains, respectively. The letter B indicates B2M.

Table S3

Correspondence between the IMGT DOMAIN numbering, the IMGT file numbering, the PDB numbering for the residues (amino acid 3-letter and one-letter abbreviations) of the domains 1. VGAMMA, 2. V-DELTA, 3. G-ALPHA1-LIKE and G-ALPHA2-LIKE of the PDB entry 4lh4 (IMGT/3Dstructure-DB card for 4lh4, https://www.imgt.org/3Dstructure-DB/cgi/details.cgi?pdbcode=4LHU, IMGT numbering comparison).

Supplementary Figures Legends

Figure S1

T cell receptor (TR)/lipid antigen-CD1D complex. TR gamma/delta is shown (on top) in complex with CD1D presenting a lipid antigen in its grove. *In vivo*, TR is anchored in the membrane of a T cell as part of the signaling T cell receptor (TcR=TR+CD3) (Lefranc, 2014). TR is made of two chains, each comprising a variable domain (V-DOMAIN) at the N-terminal end (Lefranc et al., 2003) and a constant domain (C-DOMAIN) at the C-terminal end. The domains are V-GAMMA and C-GAMMA for the TR-GAMMA chain, V-DELTA and C-DELTA for the TR-DELTA chain. CD1D is made of the I-ALPHA -LIKE chain with two G-LIKE-DOMAIN (G-ALPHA-1-LIKE and G-ALPHA-2-LIKE) (Lefranc et al., 2005; Ehrenmann et al., 2011) and a C-LIKE-DOMAIN, non-covalently associated with the B2M (a C-LIKE-DOMAIN). The following colors were used: deep blue for TR-DELTA, magenta for TR-GAMMA,

dark salmon for CD1D, deep teal for B2M. IMGT labels are in capital letters (Lefranc, 2014). The TR/CD1D complex structure is taken from 4lhu.pdb, that reports the Crystallized Structure of 9C2 TCR bound to CD1D (Linguiti et al., 2022). The IMGT gene assignment is Homsap TRGV5-TRGJ1 [6.8.16] and Homsap TRDV1-TRDD1-TRDJ1 [7.3.14] of 9C2 TR (4lhu.pdb).

Figure S2
CDR3-IMGT nucleotide (nt) and predicted amino acid (AA) sequences retrieved from the *Camelus dromedarius* TRD cDNA clones isolated from blood (Antonacci et al. 2011).

Figure S3
CDR3-IMGT nucleotide (nt) and predicted amino acid (AA) sequences retrieved from the *Camelus dromedarius* TRD cDNA clones isolated from tonsils (Antonacci et al. 2011).

Figure S4
Sequence alignment of the investigated *C. dromedarius* TRG (**A**) and TRD (**B**) chains with human TRG and TRD chains from the crystallized structures 4lhu.pdb (Uldrich et al., 2013) and 1hxm.pdb (Xu et al., 2011). Sequence-structure pairwise alignment of the human CD1D (**C**) (from 4lhu.pdb) and B2M (**D**) (from 4lhu.pdb) with their closest homologues in *C. dromedarius*.

Figure S5
Structural Assessment of the generated 3D protein complex models.

REFERENCES

Antonacci R, Mineccia M, Lefranc M-P, Ashmaoui HME, Lanave C, Piccinni B, Pesole G, Hassanane MS, Massari S, Ciccarese S. Expression and genomic analyses of Camelus dromedarius T cell receptor delta (TRD) genes reveal a variable domain repertoire enlargement due to CDR3 diversification and somatic mutation. *Mol Immunol* (2011) doi: 10.1016/j.molimm.2011.03.011

Uldrich AP, Le Nours J, Pellicci DG, Gherardin NA, McPherson KG, Lim TR, et al. CD1dlipid antigen recognition by the gδ TCR. Nat Immunol. (2013) 14: 1137-1145.doi.org/10.1038/ni.2713

Xu B, Pizarro JC, Holmes MA, McBeth C, Groh V, Spies T, Strong RK. Crystal structure of a gammadelta T-cell receptor specific for the human MHC class I

homolog MICA. Proc Natl Acad Sci US A (2011) 108(6), 2414–9.

Lefranc M-P. Immunoglobulin and T cell receptor genes: IMGT® and the birth and rise of immunoinformatics. *Front Immunol* (2014) doi: 10.3389/fimmu.2014.00022

Lefranc M-P, Pommié C, Ruiz M, Giudicelli V, Foulquier E, Truong L, Thouvenin-Contet V, Lefranc G. IMGT unique numbering for immunoglobulin and T cell receptor variable domains and Ig superfamily V-like domains. *Dev Comp Immunol* (2003) doi: 10.1016/S0145-305X(02)00039-3

Lefranc M-P, Duprat E, Kaas Q, Tranne M, Thiriot A, Lefranc G. IMGT unique numbering for MHC groove G-DOMAIN and MHC superfamily (MhcSF) G-LIKE-DOMAIN. *Dev Comp Immunol* (2005) doi: 10.1016/j.dci.2005.03.003

Ehrenmann F, Giudicelli V, Duroux P, Lefranc M-P. IMGT/collier de perles: IMGT standardized representation of domains (IG, TR, and IgSF variable and constant domains, MH and MhSF groove domains). *Cold Spring Harb Protoc* (2011) doi: 10.1101/pdb.prot5635
