# Supplementary material for: Three-Dimensional Modeling of Camelus dromedarius T Cell Receptor Gamma (TRG)_Delta (TRD)/CD1D Complex Reveals Different Binding Interactions Depending on the TRD CDR3 Length
Source: Antibodies (Basel). 2025 May 29;14(2):46. doi: 10.3390/antib14020046 (PMC12189835; doi:10.3390/antib14020046)
Supplement: Supplementary file 1 [file antibodies-14-00046-s001.zip › antibodies-3511851-supplementary/Suppl.Mat.Fig.Tab/Table S1.pdf]

Pdb	4hlu.pdb		RTS88_SC19		RTS88_SC44		RTS88_SC54		5R1S169_SC19		5R1S169_SC44		5R1S169_SC54	
**Group1**	G	GD	G	GD	G	GD	G	GD	G	GD	G	GD	G	GD
**Group2**	D	C (A)	D	C	D	C	D	C	D	C	D	C	D	C
**Interaction Energy**	-42,08	-15,95	-16,73	**-11,93**	-14,83	-5,95	-16,56	-5,36	**-23,47**	-6,24	-10,57	-8,49	**-20,10**	-5,54
**IntraclashesGroup1**	10,82	9,75	6,91	32,94	10,44	20,58	6,20	13,93	20,82	7,69	15,79	8,14	4,91	7,24
**IntraclashesGroup2**	6,99	18,48	17,37	9,80	7,09	7,71	7,05	7,36	5,36	30,78	5,08	24,84	3,99	10,50
**Backbone Hbond**	-6,81	-1,94	-6,61	-0,26	-3,97	-0,46	-4,57	-0,92	-7,77	-1,43	-3,65	-1,70	-4,52	-0,65
**Sidechain Hbond**	-10,46	-8,06	-15,50	-6,33	-8,53	-1,63	-5,81	-6,27	-6,30	-2,72	-8,94	-5,06	-7,19	-5,11
**Van der Waals**	-35,78	-14,05	-35,43	-12,01	-22,37	-10,61	-21,39	-7,80	-33,73	-11,54	-20,73	-10,79	-17,84	-8,76
**Electrostatics**	-3,08	-0,57	-0,39	-2,23	1,77	-1,20	0,12	-0,38	-3,13	-1,25	-0,31	-1,36	-0,04	-0,49
**Solvation Polar**	39,59	15,37	50,72	14,52	28,75	14,11	24,87	11,84	44,87	15,81	27,90	14,80	19,62	12,58
**Solvation Hydrophobic**	-50,04	-18,74	-43,86	-14,35	-29,00	-13,24	-27,75	-8,89	-42,94	-12,87	-25,51	-12,62	-23,33	-10,26
**Van der Waals clashes**	0,53	0,63	7,34	0,69	1,25	0,28	0,62	0,32	2,62	0,41	0,87	0,13	1,55	0,20
**Entropy sidechain**	14,77	9,40	13,41	7,27	8,15	5,09	9,84	4,78	11,45	6,75	6,05	5,88	5,50	4,88
**Entropy mainchain**	10,31	1,96	12,11	1,10	5,93	1,10	7,57	1,69	10,19	0,94	8,37	1,37	6,32	1,90
**Torsional clash**	0,13	0,04	1,33	0,08	1,81	0,18	0,06	0,04	1,99	0,07	3,09	0,37	0,05	0,01
**Backbone clash**	2,15	2,37	15,68	1,65	2,87	2,69	2,10	0,91	7,97	1,57	3,40	2,65	1,70	1,00
**Helix dipole**	-0,25	0,14	-0,02	0,32	-0,08	0,08	0,00	0,28	0,00	0,01	0,00	-0,01	0,00	-0,04
**Electrostatic kon**	-1,09	-0,15	0,09	-0,78	0,31	-0,35	-0,20	-0,09	-0,79	-0,41	0,03	-0,17	-0,29	0,16
**Entropy Complex**	2,38	2,38	2,38	2,38	2,38	2,38	2,38	2,38	2,38	2,38	2,38	2,38	2,38	2,38
**Number of Residues**	801	801	669	669	660	660	644	644	644	644	635	635	619	619
