# Supplementary material for: Three-Dimensional Modeling of Camelus dromedarius T Cell Receptor Gamma (TRG)_Delta (TRD)/CD1D Complex Reveals Different Binding Interactions Depending on the TRD CDR3 Length
Source: Antibodies (Basel). 2025 May 29;14(2):46. doi: 10.3390/antib14020046 (PMC12189835; doi:10.3390/antib14020046)
Supplement: Supplementary file 1 [file antibodies-14-00046-s001.zip › antibodies-3511851-supplementary/Suppl.Mat.Fig.Tab/Table S2.pdf]

Protein-Protein Hydrophobic Interactions

Rasmol Jmol [help]

RTS88.SC19.pdb

Hydrophobic Interactions within 5 Angstroms

Position	Residue	Chain	Position	Residue	Chain
31	LEU	C	75	PHE	B
33	ILE	C	53	PRO	B
33	ILE	C	73	LEU	B
33	ILE	C	75	PHE	B
33	ILE	C	81	PHE	B
36	TYR	G	126	PRO	D
36	TYR	G	127	LEU	D
38	PHE	G	90	PHE	D
43	ALA	G	129	PHE	D
43	ALA	G	90	PHE	D
44	PRO	G	126	PRO	D
44	PRO	G	90	PHE	D
49	TRP	C	82	TYR	B
76	PHE	C	29	TRP	D
87	ILE	C	103	TRP	D
90	VAL	C	103	TRP	D
94	LEU	G	126	PRO	D
94	LEU	G	45	MET	D
98	TRP	G	108	TRP	D
103	TYR	G	117	TRP	D
103	TYR	G	47	PHE	D
107	TRP	G	103	TRP	D
107	TRP	G	113	ALA	D
111	PHE	G	45	MET	D
135	ALA	C	79	TRP	B
140	ALA	C	79	TRP	B
178	TRP	C	103	TRP	D
178	TRP	C	31	TYR	D
183	ILE	C	29	TRP	D
208	TRP	C	34	PRO	B
254	LEU	C	30	TYR	B
254	LEU	C	46	TYR	B
255	PRO	C	46	TYR	B
255	PRO	C	84	LEU	B
257	ALA	C	84	LEU	B
262	TYR	C	30	TYR	B

NO PROTEIN-PROTEIN DISULPHIDE BRIDGES FOUND

Protein-Protein Main Chain-Main Chain Hydrogen Bonds

Rasmol Jmol [help]

[View the original hbond output]

RTS88.SC19.pdb

DONOR				ACCEPTOR					PARAMETERS			
POS	CHAIN	RES	ATOM	POS	CHAIN	RES	ATOM	MO	Dd-a	Dh-a	A(d-H-N)	A(a-O=C)
107	G	TRP	N	112	D	GLY	O	-	3.06	2.25	138.04	78.43
108	G	ARG	N	110	D	GLN	O	-	3.33	2.82	113.81	92.34
108	G	ARG	N	111	D	LYS	O	-	2.81	1.88	159.91	111.33
108	G	ARG	N	112	D	GLY	O	-	3.13	3.13	81.02	117.98
20	B	ALA	N	137	C	GLN	O	-	2.89	9.99	999.99	999.99
118	B	GLN	N	211	C	SER	O	-	2.96	2.07	145.22	136.62
110	D	GLN	N	108	G	ARG	O	-	3.31	2.32	167.95	129.12
111	D	LYS	N	106	G	GLY	O	-	2.93	2.26	123.14	82.99
112	D	GLY	N	105	G	SER	O	-	2.83	1.86	162.80	89.09
122	D	SER	N	109	G	LYS	O	-	3.22	3.24	79.68	152.46
123	D	ARG	N	109	G	LYS	O	-	2.90	1.95	168.97	120.70

Dd-a = Distance Between Donor and Acceptor
Dh-a = Distance Between Hydrogen and Acceptor
A(d-H-N) = Angle Between Donor-H-N
A(a-O=C) = Angle Between Acceptor-O=C
MO = Multiple Occupancy
Note that angles that are undefined are written as 999.99

Protein-Protein Main Chain-Side Chain Hydrogen Bonds

Rasmol Jmol [help]

[View the original hbond output]

RTS88.SC19.pdb

DONOR				ACCEPTOR					PARAMETERS			
POS	CHAIN	RES	ATOM	POS	CHAIN	RES	ATOM	MO	Dd-a	Dh-a	A(d-H-N)	A(a-O=C)
32	G	TYR	N	107	D	ASP	OD1	-	3.34	3.11	94.32	126.00
36	G	TYR	OH	123	D	ARG	O	-	3.15	9.99	999.99	140.04
98	G	TRP	NE1	106	D	THR	O	-	3.23	2.38	155.07	130.10
108	G	ARG	NH1	117	D	TRP	O	1	2.64	1.63	155.49	120.58
108	G	ARG	NH1	117	D	TRP	O	2	2.64	3.08	55.37	120.58
109	G	LYS	NZ	100	D	TYR	O	-	2.92	9.99	999.99	164.55
111	G	PHE	N	122	D	SER	OG	-	2.97	1.98	172.38	999.99

115	C	GLN	OE1	79	B	TRP	O	1	3.46	2.64	133.21	128.26
115	C	GLN	OE1	79	B	TRP	O	2	3.46	4.39	26.23	128.26
115	C	GLN	NE2	79	B	TRP	O	1	3.00	1.96	164.54	127.26
115	C	GLN	NE2	79	B	TRP	O	2	3.00	3.70	41.73	127.26
138	C	GLY	N	51	B	HIS	NE2	-	3.24	3.23	82.09	999.99
211	C	SER	N	117	B	ASP	OD1	-	2.82	1.90	154.78	136.53
225	C	HIS	NE2	118	B	GLN	O	-	3.43	3.45	81.25	93.51
227	C	SER	OG	32	B	ARG	O	-	2.74	9.99	999.99	145.23
256	C	GLN	NE2	31	B	SER	O	1	3.22	2.24	153.81	117.65
256	C	GLN	NE2	31	B	SER	O	2	3.22	3.65	58.16	117.65
257	C	ALA	N	86	B	HIS	NE2	-	3.26	2.32	160.39	999.99
264	C	ARG	NE	118	B	GLN	O	-	3.10	3.63	51.64	122.44
264	C	ARG	NH1	118	B	GLN	O	1	3.41	3.61	70.86	86.53
264	C	ARG	NH1	118	B	GLN	O	2	3.41	3.91	53.64	86.53
264	C	ARG	NH1	118	B	GLN	OXT	1	3.43	4.10	45.31	85.26
264	C	ARG	NH1	118	B	GLN	OXT	2	3.43	3.38	84.74	85.26
264	C	ARG	NH2	118	B	GLN	O	1	3.48	3.77	66.04	86.27
264	C	ARG	NH2	118	B	GLN	O	2	3.48	4.00	53.48	86.27
264	C	ARG	NH2	118	B	GLN	OXT	1	3.03	3.44	58.43	108.36
264	C	ARG	NH2	118	B	GLN	OXT	2	3.03	2.95	83.84	108.36
30	B	TYR	OH	255	C	PRO	O	-	3.07	9.99	999.99	145.97
44	B	ASN	ND2	255	C	PRO	O	1	2.93	1.89	166.65	139.22
44	B	ASN	ND2	255	C	PRO	O	2	2.93	3.44	53.27	139.22
118	B	GLN	N	210	C	SER	OG	-	3.33	2.65	124.84	999.99
109	D	THR	OG1	107	G	TRP	O	-	2.68	9.99	999.99	140.49
113	D	ALA	N	105	G	SER	OG	-	2.85	1.87	172.23	999.99
117	D	TRP	NE1	103	G	TYR	O	-	3.09	2.68	108.52	121.29
122	D	SER	OG	111	G	PHE	O	-	3.33	9.99	999.99	119.13

Dd-a = Distance Between Donor and Acceptor
Dh-a = Distance Between Hydrogen and Acceptor
A(d-H-N) = Angle Between Donor-H-N
A(a-O=C) = Angle Between Acceptor-O=C
MO = Multiple Occupancy
Note that angles that are undefined are written as 999.99

Protein-Protein Side Chain-Side Chain Hydrogen Bonds

Rasmol **Jmol** [help]

[View the original hbond output]

RTS88.SC19.pdb

	DONOR				ACCEPTOR						PARAMETERS	
**POS**	**CHAIN**	**RES**	**ATOM**	**POS**	**CHAIN**	**RES**	**ATOM**	**MO**	**Dd-a**	**Dh-a**	**A(d-H-N)**	**A(a-O=C)**
31	G	ASN	ND2	107	D	ASP	OD2	1	2.91	3.55	45.84	999.99
31	G	ASN	ND2	107	D	ASP	OD2	2	2.91	1.87	172.79	999.99
32	G	TYR	OH	86	C	HIS	NE2	-	2.91	9.99	999.99	999.99
107	G	TRP	NE1	174	C	GLU	OE1	-	2.94	2.42	115.99	999.99
107	G	TRP	NE1	174	C	GLU	OE2	-	2.91	2.01	170.03	999.99
109	G	LYS	NZ	94	D	ASP	OD1	-	2.88	9.99	999.99	999.99

109	G	LYS	NZ	94	D	ASP	OD2	-	2.72	9.99	999.99	999.99
147	G	HIS	NE2	148	D	SER	OG	-	2.94	2.09	157.13	999.99
49	C	TRP	NE1	82	B	TYR	OH	-	3.41	3.01	108.35	999.99
54	C	GLN	NE2	72	B	ASP	OD2	1	2.92	1.93	155.35	999.99
54	C	GLN	NE2	72	B	ASP	OD2	2	2.92	3.40	54.85	999.99
57	C	THR	OG1	72	B	ASP	OD2	-	2.84	9.99	999.99	999.99
59	C	ARG	NE	72	B	ASP	OD1	-	3.11	2.45	121.56	999.99
59	C	ARG	NH2	72	B	ASP	OD1	1	2.60	1.61	155.47	999.99
59	C	ARG	NH2	72	B	ASP	OD1	2	2.60	3.34	38.08	999.99
86	C	HIS	NE2	32	G	TYR	OH	-	2.91	2.03	166.82	999.99
113	C	ASP	OD2	51	B	HIS	NE2	1	2.81	3.05	67.25	999.99
113	C	ASP	OD2	51	B	HIS	NE2	2	2.81	2.15	118.10	999.99
115	C	GLN	OE1	51	B	HIS	NE2	1	3.48	3.43	83.91	999.99
115	C	GLN	OE1	51	B	HIS	NE2	2	3.48	3.18	97.12	999.99
182	C	SER	OG	30	D	SER	OG	-	2.83	9.99	999.99	999.99
206	C	GLU	OE2	33	B	HIS	ND1	1	2.91	1.97	144.95	999.99
206	C	GLU	OE2	33	B	HIS	ND1	2	2.91	3.31	59.21	999.99
210	C	SER	OG	117	B	ASP	OD1	-	3.18	9.99	999.99	999.99
256	C	GLN	OE1	44	B	ASN	ND2	1	2.65	2.54	83.53	999.99
256	C	GLN	OE1	44	B	ASN	ND2	2	2.65	2.91	64.93	999.99
256	C	GLN	OE1	86	B	HIS	NE2	1	3.41	3.29	87.01	999.99
256	C	GLN	OE1	86	B	HIS	NE2	2	3.41	2.71	122.70	999.99
32	B	ARG	NH1	206	C	GLU	OE2	1	3.15	3.79	46.36	999.99
32	B	ARG	NH1	206	C	GLU	OE2	2	3.15	2.70	107.10	999.99
32	B	ARG	NH2	206	C	GLU	OE2	1	3.39	4.11	40.91	999.99
32	B	ARG	NH2	206	C	GLU	OE2	2	3.39	2.92	107.43	999.99
33	B	HIS	ND1	206	C	GLU	OE2	-	2.91	2.07	157.85	999.99
44	B	ASN	ND2	256	C	GLN	OE1	1	2.65	3.05	57.94	999.99
44	B	ASN	ND2	256	C	GLN	OE1	2	2.65	1.69	148.62	999.99
51	B	HIS	NE2	113	C	ASP	OD2	-	2.81	2.10	136.77	999.99
51	B	HIS	NE2	115	C	GLN	OE1	-	3.48	2.97	119.09	999.99
72	B	ASP	OD2	54	C	GLN	NE2	1	2.92	3.36	57.11	999.99
72	B	ASP	OD2	54	C	GLN	NE2	2	2.92	2.01	141.65	999.99
86	B	HIS	NE2	256	C	GLN	OE1	-	3.41	2.57	156.04	999.99
30	D	SER	OG	182	C	SER	OG	-	2.83	9.99	999.99	999.99
102	D	ARG	NH1	174	C	GLU	OE1	1	2.76	3.19	56.89	999.99
102	D	ARG	NH1	174	C	GLU	OE1	2	2.76	1.80	151.45	999.99
105	D	ARG	NH1	32	G	TYR	OH	1	3.31	2.79	109.96	999.99
105	D	ARG	NH1	32	G	TYR	OH	2	3.31	3.09	93.37	999.99
105	D	ARG	NH1	49	G	TYR	OH	1	3.23	4.18	23.19	999.99
105	D	ARG	NH1	49	G	TYR	OH	2	3.23	2.47	130.95	999.99
105	D	ARG	NH2	49	G	TYR	OH	1	2.88	3.63	38.11	999.99
105	D	ARG	NH2	49	G	TYR	OH	2	2.88	1.91	152.15	999.99
105	D	ARG	NE	83	C	ASN	OD1	-	2.92	1.89	175.05	999.99
107	D	ASP	OD2	31	G	ASN	ND2	1	2.91	2.15	126.44	999.99
107	D	ASP	OD2	31	G	ASN	ND2	2	2.91	3.29	60.45	999.99
117	D	TRP	NE1	105	G	SER	OG	-	3.35	2.90	112.38	999.99
123	D	ARG	NH2	34	G	HIS	ND1	1	2.74	2.87	72.47	999.99
123	D	ARG	NH2	34	G	HIS	ND1	2	2.74	2.07	119.63	999.99
123	D	ARG	NE	36	G	TYR	OH	-	3.02	3.71	42.13	999.99
123	D	ARG	NH1	36	G	TYR	OH	1	2.84	2.53	95.52	999.99
123	D	ARG	NH1	36	G	TYR	OH	2	2.84	3.46	44.76	999.99
148	D	SER	OG	147	G	HIS	NE2	-	2.94	9.99	999.99	999.99

Dd-a = Distance Between Donor and Acceptor
Dh-a = Distance Between Hydrogen and Acceptor

A(d-H-N) = Angle Between Donor-H-N
A(a-O=C) = Angle Between Acceptor-O=C
MO = Multiple Occupancy
Note that angles that are undefined are written as 999.99

Protein-Protein Ionic Interactions

[help]

Rasmol **Jmol**

RTS88.SC19.pdb

Ionic Interactions within 6 Angstroms

Position	Residue	Chain	Position	Residue	Chain
34	HIS	G	107	ASP	D
51	ASP	G	86	HIS	C
56	LYS	G	63	ASP	C
59	ARG	C	72	ASP	B
66	ARG	C	72	ASP	B
82	GLU	C	105	ARG	D
86	HIS	C	107	ASP	D
109	LYS	G	94	ASP	D
113	ASP	C	51	HIS	B
174	GLU	C	102	ARG	D
174	GLU	C	50	ARG	D
206	GLU	C	32	ARG	B
206	GLU	C	33	HIS	B
252	ASP	C	26	LYS	B
258	ASP	C	32	ARG	B

Protein-Protein Aromatic-Aromatic Interactions

[help]

Rasmol **Jmol**

RTS88.SC19.pdb

Aromatic-Aromatic Interactions within 4.5 and 7 Angstroms

Residue	Position	Chain	Residue	Position	Chain	D(centroid-centroid)	Dihedral Angle
38	PHE	G	90	PHE	D	6.34	78.17
49	TRP	C	82	TYR	B	6.47	154.92
76	PHE	C	29	TRP	D	5.49	23.27
103	TYR	G	47	PHE	D	5.83	109.91
178	TRP	C	103	TRP	D	6.41	31.32
262	TYR	C	30	TYR	B	4.60	157.85

NO PROTEIN-PROTEIN AROMATIC-SULPHUR INTERACTIONS FOUND

Protein-Protein Cation-Pi Interactions

Rasmol **Jmol** [help]

RTS88.SC19.pdb

Cation-Pi Interactions within 6 Angstroms

Position	Residue	Chain	Position	Residue	Chain	D(cation-Pi)	Angle
36	TYR	G	123	ARG	D	4.46	56.69
47	PHE	D	108	ARG	G	4.02	170.39
49	TYR	G	105	ARG	D	5.27	126.20
100	TYR	D	109	LYS	G	5.39	31.01
107	TRP	G	102	ARG	D	4.91	22.38
108	TRP	D	46	ARG	G	5.83	48.07
178	TRP	C	96	ARG	D	5.34	75.60
