# Supplementary material for: Three-Dimensional Modeling of Camelus dromedarius T Cell Receptor Gamma (TRG)_Delta (TRD)/CD1D Complex Reveals Different Binding Interactions Depending on the TRD CDR3 Length
Source: Antibodies (Basel). 2025 May 29;14(2):46. doi: 10.3390/antib14020046 (PMC12189835; doi:10.3390/antib14020046)
Supplement: Supplementary file 1 [file antibodies-14-00046-s001.zip › antibodies-3511851-supplementary/Suppl.Mat.Fig.Tab/Table S3.pdf]

Supplementary Table S3

Correspondence between the IMGT DOMAIN numbering, the IMGT file numbering, the PDB numbering for the residues (amino acid 3-letter and one-letter abbreviations) of the domains 1. V-GAMMA, 2. V-DELTA, 3. G-ALPHA1-LIKE and G-ALPHA2-LIKE of the PDB entry 4lh4 (IMGT/3Dstructure-DB card for 4lh4, https://www.imgt.org/3Dstructure-DB/cgi/details.cgi?pdbcode=4LHU, IMGT numbering comparison).

1. V-GAMMA

CHAIN: 4lhu_G					
IMGT DOMAIN	IMGT DOMAIN numbering [1]	IMGT file numbering	PDB numbering	Residue [2]	
V-GAMMA [D1]	7V	7	10	GLY	G
V-GAMMA [D1]	8V	8	11	THR	T
V-GAMMA [D1]	9V	9	12	LYS	K
V-GAMMA [D1]	10V	10	13	SER	S
V-GAMMA [D1]	11V	11	14	VAL	V
V-GAMMA [D1]	12V	12	15	THR	T
V-GAMMA [D1]	13V	13	16	ARG	R
V-GAMMA [D1]	14V	14	17	PRO	P
V-GAMMA [D1]	15V	15	18	THR	T
V-GAMMA [D1]	16V	16	19	ARG	R
V-GAMMA [D1]	17V	17	20	SER	S
V-GAMMA [D1]	18V	18	21	SER	S
V-GAMMA [D1]	19V	19	22	ALA	A
V-GAMMA [D1]	20V	20	23	GLU	E
V-GAMMA [D1]	21V	21	24	ILE	I
V-GAMMA [D1]	22V	22	25	THR	T
V-GAMMA [D1]	23V	23	26	CYS	C
V-GAMMA [D1]	24V	24	27	ASP	D
V-GAMMA [D1]	25V	25	28	LEU	L
V-GAMMA [D1]	26V	26	29	THR	T
V-GAMMA [D1]	27V	27	30	VAL	V
V-GAMMA [D1]	28V	28	31	ILE	I
V-GAMMA [D1]	29V	29	32	ASN	N
V-GAMMA [D1]	36V	36	33	ALA	A
V-GAMMA [D1]	37V	37	34	PHE	F
V-GAMMA [D1]	38V	38	35	TYR	Y
V-GAMMA [D1]	39V	39	36	ILE	I
V-GAMMA [D1]	40V	40	37	HIS	H
V-GAMMA [D1]	41V	41	38	TRP	W
V-GAMMA [D1]	42V	42	39	TYR	Y
V-GAMMA [D1]	43V	43	40	LEU	L
V-GAMMA [D1]	44V	44	41	HIS	H
V-GAMMA [D1]	45V	45	42	GLN	Q
V-GAMMA [D1]	46V	46	43	GLU	E
V-GAMMA [D1]	47V	47	44	GLY	G
V-GAMMA [D1]	48V	48	45	LYS	K
V-GAMMA [D1]	49V	49	46	ALA	A

V-GAMMA [D1]	50V	50	47	PRO	P
V-GAMMA [D1]	51V	51	48	GLN	Q
V-GAMMA [D1]	52V	52	49	ARG	R
V-GAMMA [D1]	53V	53	50	LEU	L
V-GAMMA [D1]	54V	54	51	LEU	L
V-GAMMA [D1]	55V	55	52	TYR	Y
V-GAMMA [D1]	56V	56	53	TYR	Y
V-GAMMA [D1]	57V	57	54	ASP	D
V-GAMMA [D1]	58V	58	55	VAL	V
V-GAMMA [D1]	59V	59	56	SER	S
V-GAMMA [D1]	62V	62	57	ASN	N
V-GAMMA [D1]	63V	63	58	SER	S
V-GAMMA [D1]	64V	64	59	LYS	K
V-GAMMA [D1]	65V	65	60	ASP	D
V-GAMMA [D1]	66V	66	61	VAL	V
V-GAMMA [D1]	67V	67	62	LEU	L
V-GAMMA [D1]	68V	68	63	GLU	E
V-GAMMA [D1]	69V	69	64	SER	S
V-GAMMA [D1]	70V	70	65	GLY	G
V-GAMMA [D1]	71V	71	66	LEU	L
V-GAMMA [D1]	72V	72	67	SER	S
V-GAMMA [D1]	73V	73	68	PRO	P
V-GAMMA [D1]	74V	74	69	GLY	G
V-GAMMA [D1]	75V	75	70	LYS	K
V-GAMMA [D1]	76V	76	71	TYR	Y
V-GAMMA [D1]	77V	77	72	TYR	Y
V-GAMMA [D1]	78V	78	73	THR	T
V-GAMMA [D1]	79V	79	74	HIS	H
V-GAMMA [D1]	80V	80	75	THR	T
V-GAMMA [D1]	81V	81	76	PRO	P
V-GAMMA [D1]	83V	83	77	ARG	R
V-GAMMA [D1]	84V	84	78	ARG	R
V-GAMMA [D1]	85V	85	79	TRP	W
V-GAMMA [D1]	86V	86	80	SER	S
V-GAMMA [D1]	87V	87	81	TRP	W
V-GAMMA [D1]	88V	88	82	ILE	I
V-GAMMA [D1]	89V	89	83	LEU	L
V-GAMMA [D1]	90V	90	84	ILE	I
V-GAMMA [D1]	91V	91	85	LEU	L
V-GAMMA [D1]	92V	92	86	ARG	R
V-GAMMA [D1]	93V	93	87	ASN	N
V-GAMMA [D1]	94V	94	88	LEU	L
V-GAMMA [D1]	95V	95	89	ILE	I
V-GAMMA [D1]	96V	96	90	GLU	E
V-GAMMA [D1]	97V	97	91	ASN	N
V-GAMMA [D1]	98V	98	92	ASP	D
V-GAMMA [D1]	99V	99	93	SER	S
V-GAMMA [D1]	100V	100	94	GLY	G

V-GAMMA [D1]	101V	101	95	VAL	V
V-GAMMA [D1]	102V	102	96	TYR	Y
V-GAMMA [D1]	103V	103	97	TYR	Y
V-GAMMA [D1]	104V	104	98	CYS	C
V-GAMMA [D1]	105V	105	99	ALA	A
V-GAMMA [D1]	106V	106	100	THR	T
V-GAMMA [D1]	107V	107	101	TRP	W
V-GAMMA [D1]	108V	108	102	ASP	D
V-GAMMA [D1]	109V	109	103	ARG	R
V-GAMMA [D1]	110V	110	104	GLY	G
V-GAMMA [D1]	111V	111	105	ASN	N
V-GAMMA [D1]	111.1V	111A	106	PRO	P
V-GAMMA [D1]	112.2V	112B	107	LYS	K
V-GAMMA [D1]	112.1V	112A	108	THR	T
V-GAMMA [D1]	112V	112	109	HIS	H
V-GAMMA [D1]	113V	113	110	TYR	Y
V-GAMMA [D1]	114V	114	111	TYR	Y
V-GAMMA [D1]	115V	115	112	LYS	K
V-GAMMA [D1]	116V	116	113	LYS	K
V-GAMMA [D1]	117V	117	114	LEU	L
V-GAMMA [D1]	118V	118	115	PHE	F
V-GAMMA [D1]	119V	119	116	GLY	G
V-GAMMA [D1]	120V	120	117	SER	S
V-GAMMA [D1]	121V	121	118	GLY	G
V-GAMMA [D1]	122V	122	119	THR	T
V-GAMMA [D1]	123V	123	120	THR	T
V-GAMMA [D1]	124V	124	121	LEU	L
V-GAMMA [D1]	125V	125	122	VAL	V
V-GAMMA [D1]	126V	126	123	VAL	V
V-GAMMA [D1]	127V	127	124	THR	T

2. V-DELTA

CHAIN: 4lhu_D					
IMGT DOMAIN	IMGT DOMAIN numbering [1]	IMGT file numbering	PDB numbering	Residue [2]	
V-DELTA [D1]	2V	2	5	GLN	Q
V-DELTA [D1]	3V	3	6	LYS	K
V-DELTA [D1]	4V	4	7	VAL	V
V-DELTA [D1]	5V	5	8	THR	T
V-DELTA [D1]	6V	6	9	GLN	Q
V-DELTA [D1]	7V	7	10	ALA	A
V-DELTA [D1]	8V	8	11	GLN	Q
V-DELTA [D1]	9V	9	12	SER	S
V-DELTA [D1]	10V	10	13	SER	S

V-DELTA [D1]	11V	11	14	VAL	V
V-DELTA [D1]	12V	12	15	SER	S
V-DELTA [D1]	13V	13	16	MET	M
V-DELTA [D1]	14V	14	17	PRO	P
V-DELTA [D1]	15V	15	18	VAL	V
V-DELTA [D1]	16V	16	19	ARG	R
V-DELTA [D1]	17V	17	20	LYS	K
V-DELTA [D1]	18V	18	21	ALA	A
V-DELTA [D1]	19V	19	22	VAL	V
V-DELTA [D1]	20V	20	23	THR	T
V-DELTA [D1]	21V	21	24	LEU	L
V-DELTA [D1]	22V	22	25	ASN	N
V-DELTA [D1]	23V	23	26	CYS	C
V-DELTA [D1]	24V	24	27	LEU	L
V-DELTA [D1]	25V	25	28	TYR	Y
V-DELTA [D1]	26V	26	29	GLU	E
V-DELTA [D1]	27V	27	30	THR	T
V-DELTA [D1]	28V	28	31	SER	S
V-DELTA [D1]	29V	29	32	TRP	W
V-DELTA [D1]	30V	30	33	TRP	W
V-DELTA [D1]	36V	36	34	SER	S
V-DELTA [D1]	37V	37	35	TYR	Y
V-DELTA [D1]	38V	38	36	TYR	Y
V-DELTA [D1]	39V	39	37	ILE	I
V-DELTA [D1]	40V	40	38	PHE	F
V-DELTA [D1]	41V	41	39	TRP	W
V-DELTA [D1]	42V	42	40	TYR	Y
V-DELTA [D1]	43V	43	41	LYS	K
V-DELTA [D1]	44V	44	42	GLN	Q
V-DELTA [D1]	45V	45	43	LEU	L
V-DELTA [D1]	46V	46	44	PRO	P
V-DELTA [D1]	47V	47	45	SER	S
V-DELTA [D1]	48V	48	46	LYS	K
V-DELTA [D1]	49V	49	47	GLU	E
V-DELTA [D1]	50V	50	48	MET	M
V-DELTA [D1]	51V	51	49	ILE	I
V-DELTA [D1]	52V	52	50	PHE	F
V-DELTA [D1]	53V	53	51	LEU	L
V-DELTA [D1]	54V	54	52	ILE	I
V-DELTA [D1]	55V	55	53	ARG	R
V-DELTA [D1]	56V	56	54	GLN	Q

V-DELTA [D1]	57V	57	55	GLY	G
V-DELTA [D1]	65V	65	56	SER	S
V-DELTA [D1]	66V	66	57	ASP	D
V-DELTA [D1]	67V	67	58	GLU	E
V-DELTA [D1]	68V	68	59	GLN	Q
V-DELTA [D1]	69V	69	60	ASN	N
V-DELTA [D1]	70V	70	61	ALA	A
V-DELTA [D1]	71V	71	62	LYS	K
V-DELTA [D1]	72V	72	63	SER	S
V-DELTA [D1]	74V	74	64	GLY	G
V-DELTA [D1]	75V	75	65	ARG	R
V-DELTA [D1]	76V	76	66	TYR	Y
V-DELTA [D1]	77V	77	67	SER	S
V-DELTA [D1]	78V	78	68	VAL	V
V-DELTA [D1]	79V	79	69	ASN	N
V-DELTA [D1]	80V	80	70	PHE	F
V-DELTA [D1]	81V	81	71	LYS	K
V-DELTA [D1]	82V	82	72	LYS	K
V-DELTA [D1]	83V	83	73	ALA	A
V-DELTA [D1]	84V	84	74	ALA	A
V-DELTA [D1]	85V	85	75	LYS	K
V-DELTA [D1]	86V	86	76	SER	S
V-DELTA [D1]	87V	87	77	VAL	V
V-DELTA [D1]	88V	88	78	ALA	A
V-DELTA [D1]	89V	89	79	LEU	L
V-DELTA [D1]	90V	90	80	THR	T
V-DELTA [D1]	91V	91	81	ILE	I
V-DELTA [D1]	92V	92	82	SER	S
V-DELTA [D1]	93V	93	83	ALA	A
V-DELTA [D1]	94V	94	84	LEU	L
V-DELTA [D1]	95V	95	85	GLN	Q
V-DELTA [D1]	96V	96	86	LEU	L
V-DELTA [D1]	97V	97	87	GLU	E
V-DELTA [D1]	98V	98	88	ASP	D
V-DELTA [D1]	99V	99	89	SER	S
V-DELTA [D1]	100V	100	90	ALA	A
V-DELTA [D1]	101V	101	91	LYS	K
V-DELTA [D1]	102V	102	92	TYR	Y
V-DELTA [D1]	103V	103	93	PHE	F
V-DELTA [D1]	104V	104	94	CYS	C
V-DELTA [D1]	105V	105	95	ALA	A

V-DELTA [D1]	106V	106	96	LEU	L
V-DELTA [D1]	107V	107	97	GLY	G
V-DELTA [D1]	108V	108	98	ASP	D
V-DELTA [D1]	109V	109	99	PRO	P
V-DELTA [D1]	110V	110	100	GLY	G
V-DELTA [D1]	111V	111	101	GLY	G
V-DELTA [D1]	112.1V	112A	102	LEU	L
V-DELTA [D1]	112V	112	103	ASN	N
V-DELTA [D1]	113V	113	104	THR	T
V-DELTA [D1]	114V	114	105	ASP	D
V-DELTA [D1]	115V	115	106	LYS	K
V-DELTA [D1]	116V	116	107	LEU	L
V-DELTA [D1]	117V	117	108	ILE	I
V-DELTA [D1]	118V	118	109	PHE	F
V-DELTA [D1]	119V	119	110	GLY	G
V-DELTA [D1]	120V	120	111	LYS	K
V-DELTA [D1]	121V	121	112	GLY	G
V-DELTA [D1]	122V	122	113	THR	T
V-DELTA [D1]	123V	123	114	ARG	R
V-DELTA [D1]	124V	124	115	VAL	V
V-DELTA [D1]	125V	125	116	THR	T
V-DELTA [D1]	126V	126	117	VAL	V
V-DELTA [D1]	127V	127	118	GLU	E
V-DELTA [D1]	128V	128	119	PRO	P

3. G-ALPHA1-LIKE and G-ALPHA2-LIKE

CHAIN: 4lhu_A					
IMGT DOMAIN	IMGT DOMAIN numbering	IMGT file numbering	PDB numbering	Residue	
G-ALPHA1-LIKE [D1]	1G	1	6	ARG	R
G-ALPHA1-LIKE [D1]	2G	2	7	LEU	L
G-ALPHA1-LIKE [D1]	3G	3	8	PHE	F
G-ALPHA1-LIKE [D1]	4G	4	9	PRO	P
G-ALPHA1-LIKE [D1]	5G	5	10	LEU	L
G-ALPHA1-LIKE [D1]	6G	6	11	ARG	R
G-ALPHA1-LIKE [D1]	7G	7	12	CYS	C
G-ALPHA1-LIKE [D1]	8G	8	13	LEU	L
G-ALPHA1-LIKE [D1]	9G	9	14	GLN	Q
G-ALPHA1-LIKE [D1]	10G	10	15	ILE	I
G-ALPHA1-LIKE [D1]	11G	11	16	SER	S
G-ALPHA1-LIKE [D1]	12G	12	17	SER	S
G-ALPHA1-LIKE [D1]	13G	13	18	PHE	F
G-ALPHA1-LIKE [D1]	14G	14	19	ALA	A
G-ALPHA1-LIKE [D1]	15G	15	20	ASN	N
G-ALPHA1-LIKE [D1]	16G	16	21	SER	S
G-ALPHA1-LIKE [D1]	18G	18	22	SER	S
G-ALPHA1-LIKE [D1]	19G	19	23	TRP	W
G-ALPHA1-LIKE [D1]	20G	20	24	THR	T
G-ALPHA1-LIKE [D1]	21G	21	25	ARG	R
G-ALPHA1-LIKE [D1]	22G	22	26	THR	T
G-ALPHA1-LIKE [D1]	23G	23	27	ASP	D
G-ALPHA1-LIKE [D1]	24G	24	28	GLY	G
G-ALPHA1-LIKE [D1]	25G	25	29	LEU	L
G-ALPHA1-LIKE [D1]	26G	26	30	ALA	A
G-ALPHA1-LIKE [D1]	27G	27	31	TRP	W
G-ALPHA1-LIKE [D1]	28G	28	32	LEU	L
G-ALPHA1-LIKE [D1]	29G	29	33	GLY	G
G-ALPHA1-LIKE [D1]	30G	30	34	GLU	E
G-ALPHA1-LIKE [D1]	31G	31	35	LEU	L
G-ALPHA1-LIKE [D1]	32G	32	36	GLN	Q
G-ALPHA1-LIKE [D1]	33G	33	37	THR	T
G-ALPHA1-LIKE [D1]	34G	34	38	HIS	H
G-ALPHA1-LIKE [D1]	35G	35	39	SER	S
G-ALPHA1-LIKE [D1]	36G	36	40	TRP	W
G-ALPHA1-LIKE [D1]	37G	37	41	SER	S
G-ALPHA1-LIKE [D1]	38G	38	42	ASN	N

G-ALPHA1-LIKE [D1]	39G	39	43	ASP	D
G-ALPHA1-LIKE [D1]	42G	42	44	SER	S
G-ALPHA1-LIKE [D1]	43G	43	45	ASP	D
G-ALPHA1-LIKE [D1]	44G	44	46	THR	T
G-ALPHA1-LIKE [D1]	45G	45	47	VAL	V
G-ALPHA1-LIKE [D1]	46G	46	48	ARG	R
G-ALPHA1-LIKE [D1]	47G	47	49	SER	S
G-ALPHA1-LIKE [D1]	48G	48	50	LEU	L
G-ALPHA1-LIKE [D1]	49G	49	51	LYS	K
G-ALPHA1-LIKE [D1]	50G	50	52	PRO	P
G-ALPHA1-LIKE [D1]	51G	51	53	TRP	W
G-ALPHA1-LIKE [D1]	52G	52	54	SER	S
G-ALPHA1-LIKE [D1]	53G	53	55	GLN	Q
G-ALPHA1-LIKE [D1]	54G	54	56	GLY	G
G-ALPHA1-LIKE [D1]	54AG	54A	57	THR	T
G-ALPHA1-LIKE [D1]	55G	55	58	PHE	F
G-ALPHA1-LIKE [D1]	56G	56	59	SER	S
G-ALPHA1-LIKE [D1]	57G	57	60	ASP	D
G-ALPHA1-LIKE [D1]	58G	58	61	GLN	Q
G-ALPHA1-LIKE [D1]	59G	59	62	GLN	Q
G-ALPHA1-LIKE [D1]	60G	60	63	TRP	W
G-ALPHA1-LIKE [D1]	61G	61	64	GLU	E
G-ALPHA1-LIKE [D1]	62G	62	65	THR	T
G-ALPHA1-LIKE [D1]	63G	63	66	LEU	L
G-ALPHA1-LIKE [D1]	64G	64	67	GLN	Q
G-ALPHA1-LIKE [D1]	65G	65	68	HIS	H
G-ALPHA1-LIKE [D1]	66G	66	69	ILE	I
G-ALPHA1-LIKE [D1]	67G	67	70	PHE	F
G-ALPHA1-LIKE [D1]	68G	68	71	ARG	R
G-ALPHA1-LIKE [D1]	69G	69	72	VAL	V
G-ALPHA1-LIKE [D1]	70G	70	73	TYR	Y
G-ALPHA1-LIKE [D1]	71G	71	74	ARG	R
G-ALPHA1-LIKE [D1]	72G	72	75	SER	S
G-ALPHA1-LIKE [D1]	73G	73	76	SER	S
G-ALPHA1-LIKE [D1]	74G	74	77	PHE	F
G-ALPHA1-LIKE [D1]	75G	75	78	THR	T
G-ALPHA1-LIKE [D1]	76G	76	79	ARG	R
G-ALPHA1-LIKE [D1]	77G	77	80	ASP	D
G-ALPHA1-LIKE [D1]	78G	78	81	VAL	V
G-ALPHA1-LIKE [D1]	79G	79	82	LYS	K

G-ALPHA1-LIKE [D1]	80G	80	83	GLU	E
G-ALPHA1-LIKE [D1]	81G	81	84	PHE	F
G-ALPHA1-LIKE [D1]	82G	82	85	ALA	A
G-ALPHA1-LIKE [D1]	83G	83	86	LYS	K
G-ALPHA1-LIKE [D1]	84G	84	87	MET	M
G-ALPHA1-LIKE [D1]	85G	85	88	LEU	L
G-ALPHA1-LIKE [D1]	86G	86	89	ARG	R
G-ALPHA1-LIKE [D1]	87G	87	90	LEU	L
G-ALPHA1-LIKE [D1]	88G	88	91	SER	S
G-ALPHA2-LIKE [D2]	1G	1001	92	TYR	Y
G-ALPHA2-LIKE [D2]	2G	1002	93	PRO	P
G-ALPHA2-LIKE [D2]	3G	1003	94	LEU	L
G-ALPHA2-LIKE [D2]	4G	1004	95	GLU	E
G-ALPHA2-LIKE [D2]	5G	1005	96	LEU	L
G-ALPHA2-LIKE [D2]	6G	1006	97	GLN	Q
G-ALPHA2-LIKE [D2]	7G	1007	98	VAL	V
G-ALPHA2-LIKE [D2]	8G	1008	99	SER	S
G-ALPHA2-LIKE [D2]	9G	1009	100	ALA	A
G-ALPHA2-LIKE [D2]	10G	1010	101	GLY	G
G-ALPHA2-LIKE [D2]	11G	1011	102	CYS	C
G-ALPHA2-LIKE [D2]	12G	1012	103	GLU	E
G-ALPHA2-LIKE [D2]	13G	1013	104	VAL	V
G-ALPHA2-LIKE [D2]	14G	1014	105	HIS	H
G-ALPHA2-LIKE [D2]	15G	1015	106	PRO	P
G-ALPHA2-LIKE [D2]	16G	1016	107	GLY	G
G-ALPHA2-LIKE [D2]	18G	1018	108	ASN	N
G-ALPHA2-LIKE [D2]	19G	1019	109	ALA	A
G-ALPHA2-LIKE [D2]	20G	1020	110	SER	S
G-ALPHA2-LIKE [D2]	21G	1021	111	ASN	N
G-ALPHA2-LIKE [D2]	22G	1022	112	ASN	N
G-ALPHA2-LIKE [D2]	23G	1023	113	PHE	F
G-ALPHA2-LIKE [D2]	24G	1024	114	PHE	F
G-ALPHA2-LIKE [D2]	25G	1025	115	HIS	H
G-ALPHA2-LIKE [D2]	26G	1026	116	VAL	V
G-ALPHA2-LIKE [D2]	27G	1027	117	ALA	A
G-ALPHA2-LIKE [D2]	28G	1028	118	PHE	F
G-ALPHA2-LIKE [D2]	29G	1029	119	GLN	Q
G-ALPHA2-LIKE [D2]	30G	1030	120	GLY	G
G-ALPHA2-LIKE [D2]	31G	1031	121	LYS	K
G-ALPHA2-LIKE [D2]	32G	1032	122	ASP	D

G-ALPHA2-LIKE [D2]	33G	1033	123	ILE	I
G-ALPHA2-LIKE [D2]	34G	1034	124	LEU	L
G-ALPHA2-LIKE [D2]	35G	1035	125	SER	S
G-ALPHA2-LIKE [D2]	36G	1036	126	PHE	F
G-ALPHA2-LIKE [D2]	37G	1037	127	GLN	Q
G-ALPHA2-LIKE [D2]	38G	1038	128	GLY	G
G-ALPHA2-LIKE [D2]	43G	1043	129	THR	T
G-ALPHA2-LIKE [D2]	44G	1044	130	SER	S
G-ALPHA2-LIKE [D2]	45G	1045	131	TRP	W
G-ALPHA2-LIKE [D2]	46G	1046	132	GLU	E
G-ALPHA2-LIKE [D2]	47G	1047	133	PRO	P
G-ALPHA2-LIKE [D2]	48G	1048	134	THR	T
G-ALPHA2-LIKE [D2]	49G	1049	135	GLN	Q
G-ALPHA2-LIKE [D2]	49.1G	1049A	136	GLU	E
G-ALPHA2-LIKE [D2]	49.2G	1049B	137	ALA	A
G-ALPHA2-LIKE [D2]	49.3G	1049C	138	PRO	P
G-ALPHA2-LIKE [D2]	50G	1050	139	LEU	L
G-ALPHA2-LIKE [D2]	51G	1051	140	TRP	W
G-ALPHA2-LIKE [D2]	52G	1052	141	VAL	V
G-ALPHA2-LIKE [D2]	53G	1053	142	ASN	N
G-ALPHA2-LIKE [D2]	54G	1054	143	LEU	L
G-ALPHA2-LIKE [D2]	55G	1055	144	ALA	A
G-ALPHA2-LIKE [D2]	56G	1056	145	ILE	I
G-ALPHA2-LIKE [D2]	57G	1057	146	GLN	Q
G-ALPHA2-LIKE [D2]	58G	1058	147	VAL	V
G-ALPHA2-LIKE [D2]	59G	1059	148	LEU	L
G-ALPHA2-LIKE [D2]	60G	1060	149	ASN	N
G-ALPHA2-LIKE [D2]	61G	1061	150	GLN	Q
G-ALPHA2-LIKE [D2]	61AG	1061A	151	ASP	D
G-ALPHA2-LIKE [D2]	61BG	1061B	152	LYS	K
G-ALPHA2-LIKE [D2]	62G	1062	153	TRP	W
G-ALPHA2-LIKE [D2]	63G	1063	154	THR	T
G-ALPHA2-LIKE [D2]	64G	1064	155	ARG	R
G-ALPHA2-LIKE [D2]	65G	1065	156	GLU	E
G-ALPHA2-LIKE [D2]	66G	1066	157	THR	T
G-ALPHA2-LIKE [D2]	67G	1067	158	VAL	V
G-ALPHA2-LIKE [D2]	68G	1068	159	GLN	Q
G-ALPHA2-LIKE [D2]	69G	1069	160	TRP	W
G-ALPHA2-LIKE [D2]	70G	1070	161	LEU	L
G-ALPHA2-LIKE [D2]	71G	1071	162	LEU	L

G-ALPHA2-LIKE [D2]	72G	1072	163	ASN	N
G-ALPHA2-LIKE [D2]	72AG	1072A	164	GLY	G
G-ALPHA2-LIKE [D2]	73G	1073	165	THR	T
G-ALPHA2-LIKE [D2]	74G	1074	166	CYS	C
G-ALPHA2-LIKE [D2]	75G	1075	167	PRO	P
G-ALPHA2-LIKE [D2]	76G	1076	168	GLN	Q
G-ALPHA2-LIKE [D2]	77G	1077	169	PHE	F
G-ALPHA2-LIKE [D2]	78G	1078	170	VAL	V
G-ALPHA2-LIKE [D2]	79G	1079	171	SER	S
G-ALPHA2-LIKE [D2]	80G	1080	172	GLY	G
G-ALPHA2-LIKE [D2]	81G	1081	173	LEU	L
G-ALPHA2-LIKE [D2]	82G	1082	174	LEU	L
G-ALPHA2-LIKE [D2]	83G	1083	175	GLU	E
G-ALPHA2-LIKE [D2]	84G	1084	176	SER	S
G-ALPHA2-LIKE [D2]	85G	1085	177	GLY	G
G-ALPHA2-LIKE [D2]	86G	1086	178	LYS	K
G-ALPHA2-LIKE [D2]	87G	1087	179	SER	S
G-ALPHA2-LIKE [D2]	88G	1088	180	GLU	E
G-ALPHA2-LIKE [D2]	89G	1089	181	LEU	L
G-ALPHA2-LIKE [D2]	90G	1090	182	LYS	K
G-ALPHA2-LIKE [D2]	91G	1091	183	LYS	K
G-ALPHA2-LIKE [D2]	92G	1092	184	GLN	Q
