# Supplementary figures and images for: Three-Dimensional Modeling of Camelus dromedarius T Cell Receptor Gamma (TRG)_Delta (TRD)/CD1D Complex Reveals Different Binding Interactions Depending on the TRD CDR3 Length
Source: Antibodies (Basel). 2025 May 29;14(2):46. doi: 10.3390/antib14020046 (PMC12189835; doi:10.3390/antib14020046)

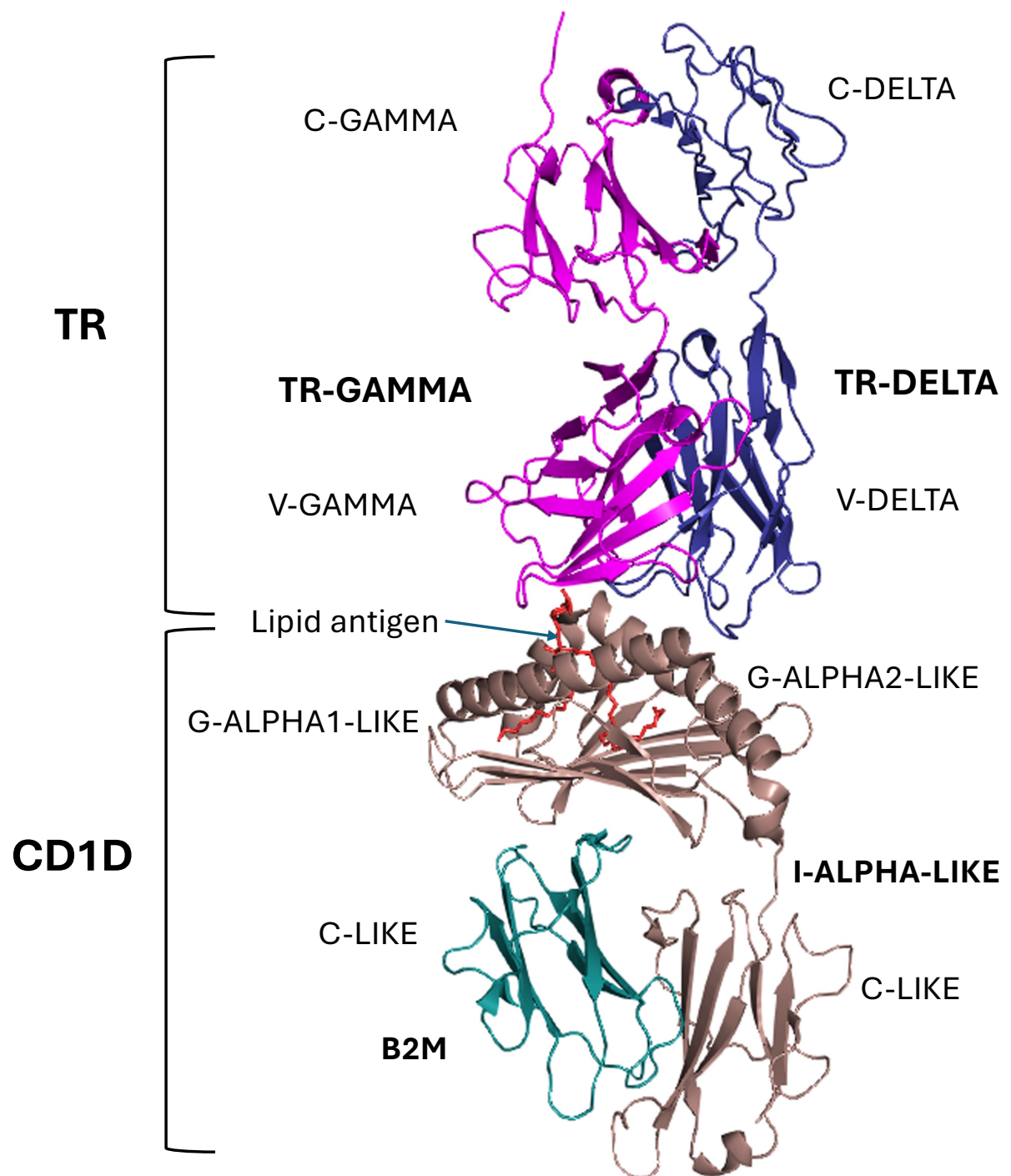

Supplement: Supplementary file 1 [file antibodies-14-00046-s001.zip › antibodies-3511851-supplementary/Suppl.Mat.Fig.Tab/Figure S1.pdf]

A

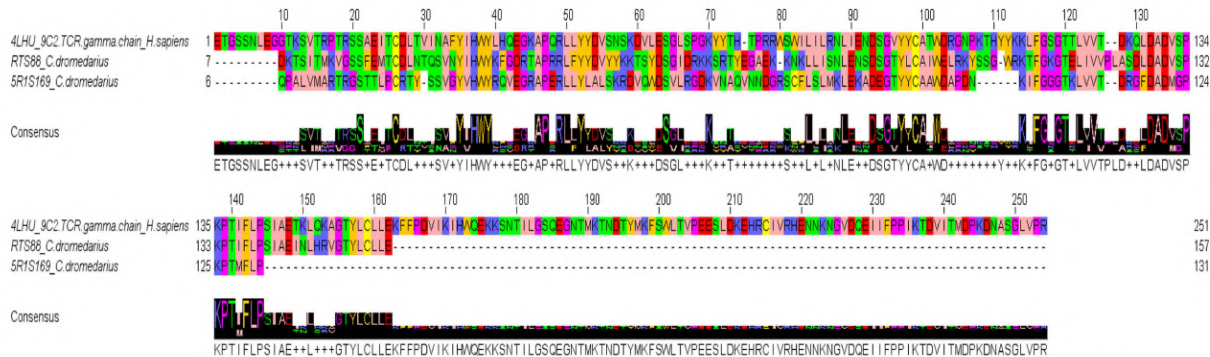

B

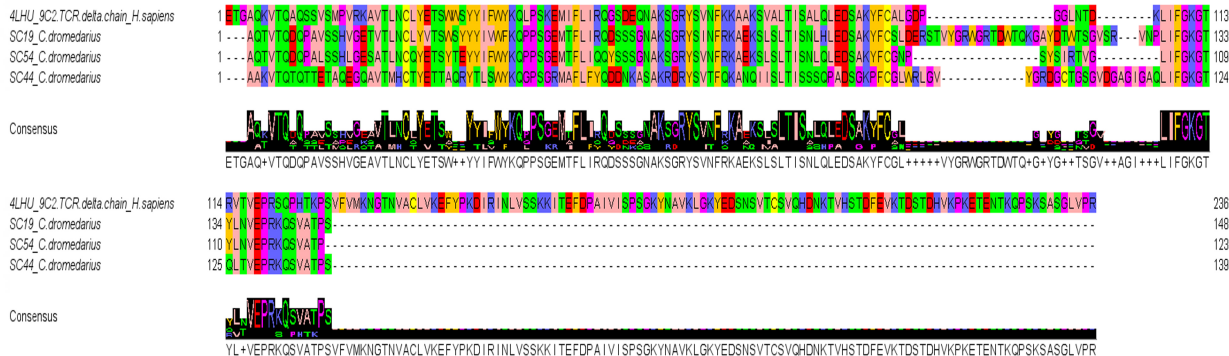

C

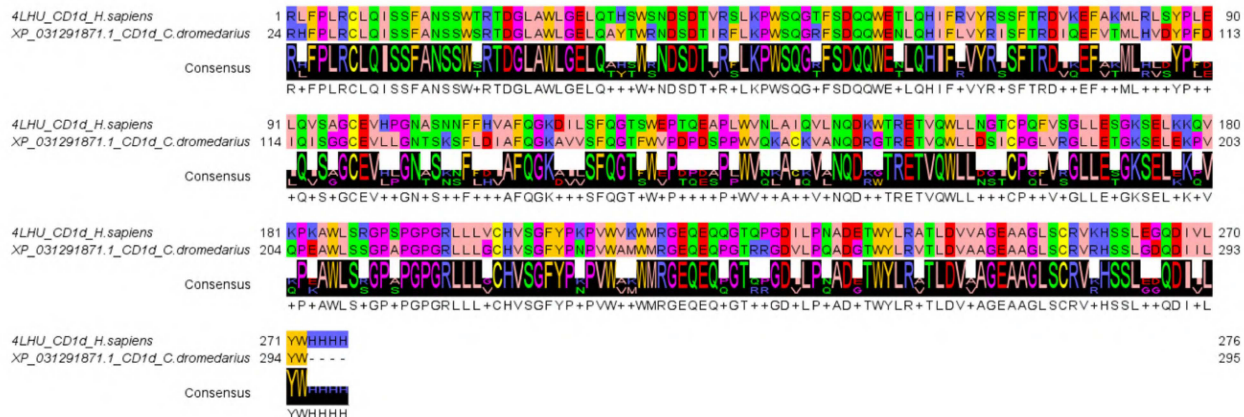

D

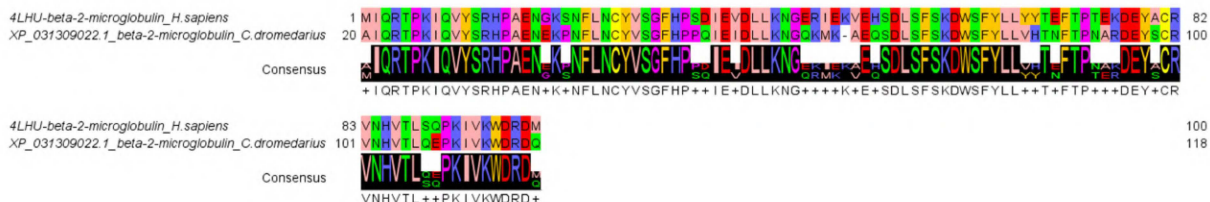

Supplement: Supplementary file 1 [file antibodies-14-00046-s001.zip › antibodies-3511851-supplementary/Suppl.Mat.Fig.Tab/Figure S4 A, B, C, D copy.ok.pdf]
